# Blood pressure stratification using photoplethysmography and light gradient boosting machine

**DOI:** 10.3389/fphys.2023.1072273

**Published:** 2023-02-20

**Authors:** Xudong Hu, Shimin Yin, Xizhuang Zhang, Carlo Menon, Cheng Fang, Zhencheng Chen, Mohamed Elgendi, Yongbo Liang

**Affiliations:** ^1^ School of Life and Environmental Sciences, Guilin University of Electronic Technology, Guilin, China; ^2^ School of Electronic Engineering and Automation, Guilin University of Electronic Technology, Guilin, China; ^3^ Biomedical and Mobile Health Technology Lab, ETH Zurich, Zurich, Switzerland; ^4^ Guangxi Colleges and Universities Key Laboratory of Biomedical Sensors and Intelligent Instruments, Guilin, China; ^5^ Guangxi Engineering Technology Research Center of Human Physiological Information Noninvasive Detection, Guilin, China

**Keywords:** blood pressure monitoring, photoplethysmography, machine learning, Optuna-tuned LightGBM, hypertension evaluation, wearable devices

## Abstract

**Introduction:** Globally, hypertension (HT) is a substantial risk factor for cardiovascular disease and mortality; hence, rapid identification and treatment of HT is crucial. In this study, we tested the light gradient boosting machine (LightGBM) machine learning method for blood pressure stratification based on photoplethysmography (PPG), which is used in most wearable devices.

**Methods:** We used 121 records of PPG and arterial blood pressure (ABP) signals from the Medical Information Mart for Intensive Care III public database. PPG, velocity plethysmography, and acceleration plethysmography were used to estimate blood pressure; the ABP signals were used to determine the blood pressure stratification categories. Seven feature sets were established and used to train the Optuna-tuned LightGBM model. Three trials compared normotension (NT) vs. prehypertension (PHT), NT vs. HT, and NT + PHT vs. HT.

**Results:** The F1 scores for these three classification trials were 90.18%, 97.51%, and 92.77%, respectively. The results showed that combining multiple features from PPG and its derivative led to a more accurate classification of HT classes than using features from only the PPG signal.

**Discussion:** The proposed method showed high accuracy in stratifying HT risks, providing a noninvasive, rapid, and robust method for the early detection of HT, with promising applications in the field of wearable cuffless blood pressure measurement.

## 1 Introduction

Globally, cardiovascular disease (CVD) is the main cause of mortality (Al-Makki et al., 2022). The World Health Organization predicts that the death rate from CVD will increase from 246 per million people in 2015 to 264 per million people in 2030 (Roth et al., 2015; Ribas Ripoll et al., 2016). Over 25% of adults worldwide suffer from hypertension (HT), which is the major cause of CVD events and mortality. Since HT typically has no symptoms or indicators in its early stages, it is referred to as the silent killer (Mensah, 2019; Polak-Iwaniuk et al., 2019). Consequently, many people are unaware that they are suffering from high blood pressure and are not treated in a timely manner. Early diagnosis, treatment, and management of HT are crucial for preventing and treating CVDs.

Blood pressure measurement methods can be invasive or noninvasive. The former is referred to as arterial puncture measurement as it requires making a puncture or incision in a blood vessel. Thus, it is only suitable for critically ill patients and not daily monitoring. Korotkoff’s sound and oscillometric methods, which require the use of an upper arm cuff and use the cuff pressure and release process to identify systolic and diastolic blood pressure levels, are the most widely used noninvasive blood pressure measuring techniques. Medical personnel and patients appreciate the reliability of these two approaches (Martínez et al., 2018). However, the Korotkoff’s sound and oscillographic methods can only provide intermittent blood pressure measurements, with a 2-min interval between measurements. Therefore, they are not suitable for real-time prediction and assessment of HT (Slapničar et al., 2019). Developing a method to determine the classification of HT that can be applied continuously and for which the results can be obtained instantly has become a popular topic of research in the digital health industry (Hosanee et al., 2020).

In the last 10 years, photoplethysmography (PPG), a noninvasive technique for monitoring changes in microvascular blood volume, has been the method most frequently studied (Elgendi et al., 2019; Park et al., 2022). Research on PPG is typically focused on two aspects: 1) the PPG-based pulse arrival time (PAT) and 2) PPG-based time and frequency domain parameters. The PAT of the former method is calculated from the PPG and the electrocardiographic (ECG) signal. However, this method requires simultaneous measurement at two different sites on the body, which can be inconvenient and challenging for some patients (Elgendi, 2020). The latter approach models PPG time and frequency domain factors to determine blood pressure. However, the analysis and information extraction of the PPG waveform morphology is extremely demanding in terms of calculating time-frequency domain parameters. This method is extremely sensitive to noise and requires high-quality PPG signals, for example, with a high sample rate and accurate sampling, which limits its wide application.

A number of scholars have conducted research to overcome these problems. In 2020, Tjahjadi et al. classified blood pressure values using the K-nearest neighbors (KNN) method based on PPG. The suggested technique improves classification accuracy without the PPG waveform shape (Tjahjadi and Ramli, 2020). However, before KNN is applied to the dataset, feature scaling (standardization and normalization) is needed, adding a data preprocessing step. Recently, deep learning approaches have been effectively used to address this issue. Sun et al. (2021) suggested a deep learning approach for classifying blood pressure using a PPG signal as well as its first and second derivative signals. They employed a convolutional neural network based on the Hilbert–Huang transform. Compared to feature extraction approaches, the method obtained greater accuracy in HT risk categorization and demonstrated that PPG derivatives include crucial information on blood pressure. However, deep learning methods require training using large-scale data, and the training time consumption is often greater than 5 h (Tjahjadi and Ramli, 2020).

We sought to address these limitations while reducing the impact of morphological methods on model stability, and we proposed a method to classify blood pressure using Tsfresh and an Optuna-tuned light gradient boosting machine (LightGBM) based on PPG and its derivatives. The suggested technique worked well for classifying blood pressure in real time. Tsfresh is a *Python* tool for time series feature extraction. Optuna-tuned LightGBM has the advantages of better model training accuracy and lower memory consumption in comparison to traditional classification algorithms, making it suitable for use in wearable devices.

The key contributions of this study are:

1) We classified blood pressure as normotension (NT), prehypertension (PHT), and HT according to the seventh report of the United States Joint National Committee on Prevention, Detection, Evaluation and Treatment of High Blood Pressure (JNC7). Our proposed method allows users to instantly know their blood pressure condition and provides a warning system for patients that may possibly have hypertension.
2) With our proposed method, only one physiological pulse wave signal is needed, and our research shows that PPG and its derivatives can be used to predict blood pressure in place of the combination of ECG and PPG. This advantage has great application potential in wearable devices, as, in general, traditional smart bracelets and smart watches can easily obtain a stable PPG signal.
3) Our proposed method has no requirement in terms of PPG signal quality and does not require extraction of pulse wave morphological features.
4) To obtain a shorter training period and lower computational complexity, our proposed method uses machine learning rather than deep learning.

## 2 Materials and methods

### 2.1 Data acquisition

In this study, arterial blood pressure (ABP) signals, measured with a catheter in the radial artery, were used to categorize the PPG signals based on the blood pressure values. They were obtained from the Medical Information Mart for Intensive Care III (MIMIC-III) database (Saeed et al., 2011; Johnson et al., 2016), a large, free, public database that contains complex-parameter recordings of more than 40,000 intensive care unit (ICU) patients, including laboratory test data, demographic information data, and physical measurements. In this paper, only the PPG and ABP signal data were used as the original data source. To correctly obtain blood pressure labels, we excluded recordings, such as missing peaks, double-peaked pulses, and no signal (Liang et al., 2018a; Liang et al., 2019). Ultimately, the recordings of 121 subjects were collected, each lasting 120 s, with a sampling frequency of 125 Hz.

### 2.2 Signal preprocessing

**FIGURE 1:**
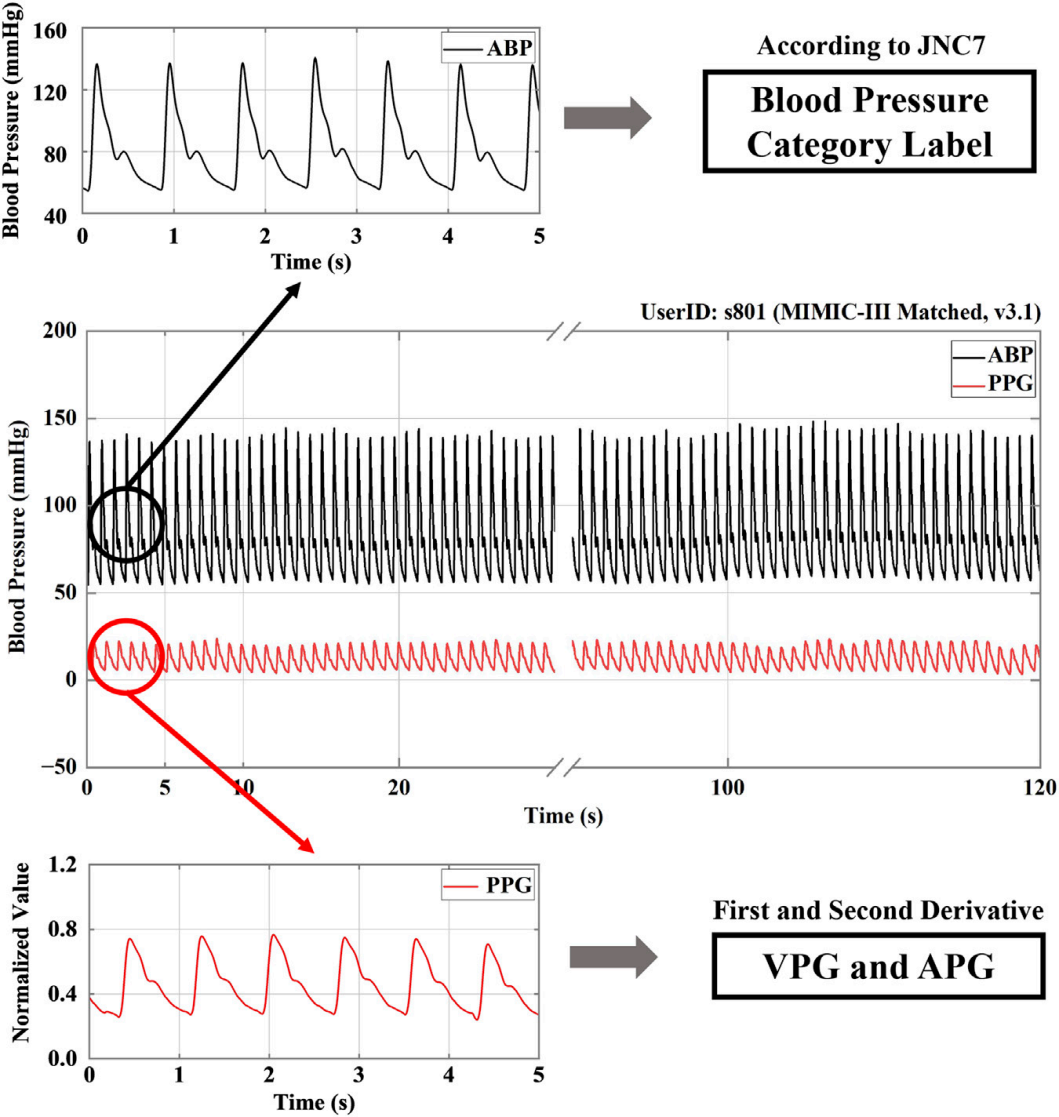
A signal processing structure. Note: ABP stands for arterial blood pressure; PPG, VPG, and APG refer to photoplethysmography, velocity plethysmography, and acceleration plethysmography, respectively. JNC7 stands for the seventh report of the United States Joint National Committee on Prevention, Detection, Evaluation and Treatment of High Blood Pressure.

Each recording included ABP and PPG signals as the target source and prediction source, respectively, and the recording was divided into 5-s segments. Next, to create the training data, we adopted the signal function extreme value search algorithm, which was mainly used to detect the peak and trough points in the ABP signal and to extract the diastolic blood pressure (DBP) and systolic blood pressure (SBP). According to JNC7 (Chobanian et al., 2003), the blood pressure conditions were classified as NT, PHT, or HT. Figure 1 illustrates the structure of the signal processing.

**FIGURE 2:**
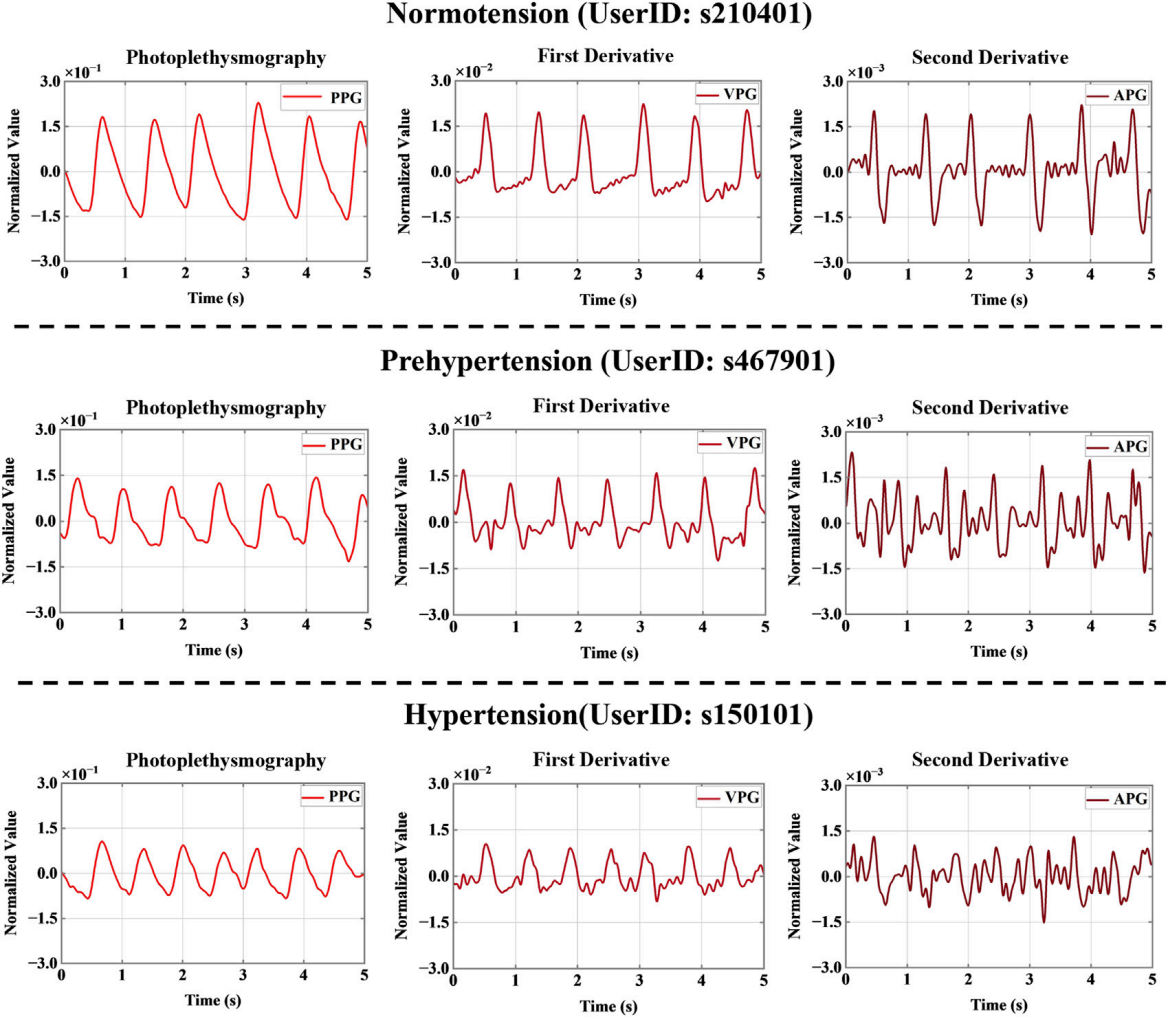
Signal derivatives for three different blood pressure categories. Note: PPG, VPG, and APG refer to photoplethysmography, velocity plethysmography, and acceleration plethysmography, respectively.

The PPG signal was then processed by primary and secondary differentiation to obtain its first and second derivative signals, which represent velocity plethysmography (VPG) and acceleration plethysmography (APG), respectively (Elgendi et al., 2018). Because a signal collected manually or by machine is inevitably subject to disturbance by the environment and other factors, such as circuit interference, resulting in the presence of various kinds of noise in the collected signal, noise reduction was an essential part of signal processing. Current noise reduction methods include filters, digital filters, Fourier transforms, wavelet transforms, *etc.* In the study discussed in this paper, the noise was reduced using a 0.5–10 Hz Butterworth bandpass filter. Then, to map the data to the same scale, the filtered PPG signals were mean-variance normalized. Figure 2 shows the PPG, VPG, and APG waveforms for the three different blood pressure categories.

### 2.3 Feature extraction

Previous PPG research faced challenges in terms of the effective location and extraction of feature points, and the traditional manual method of extracting signal feature points did not work well for poor-quality PPG signals (Chan et al., 2019). In this study, the *Python* package Tsfresh was used to extract signal segment characteristics (Christ et al., 2018). Tsfresh is a feature engineering tool for relational databases dealing with time series. It provides 77 time series characterization methods, with a total of 794 time series features that can be computed with different parameters (Gedam and Paul, 2020; Hunter et al., 2020). In real-world scenarios, time series often contain noise and redundant or irrelevant information. To avoid extracting features with low relevance, we obtained *p*-values by performing univariate feature significance tests, which were then evaluated using the Benjamini-Hochberg procedure, retaining the features with high correlation with the classification label in order to identify the features that best explained the trend (Li and Barber, 2019; Phillips et al., 2021).

Multiple temporal subsegments with classification labels *y* were simultaneously imported into the Tsfresh function, and the numerical features of the temporal subsegments were extracted; thus, 794 features were extracted for each temporal subsegment (Simjanoska et al., 2020). We then filtered out the features that did not have a significant impact on the recognition result. A total of 189 features were obtained for each PPG timing subsegment, 200 features for each VPG timing subsegment, and 190 features for each APG timing subsegment. All the features can be found in the Supplementary Material.

The following are some of the Tsfresh-calculated features:

1) Absolute energy: this term refers to the absolute energy of the time series and is the sum of the squared values.

$$E=\underset{i=1,\ldots ,n}{\sum }x_{i}^{2}$$ (1)

2) Continuous wavelet transform (CWT) coefficients: these are used to perform a CWT on the Ricker wavelet.

$$cwt=\frac{2}{\sqrt{3a}\pi^{\frac{1}{4}}}\left(1-\frac{x^{2}}{a^{2}}\right)\exp\left(-\frac{x^{2}}{2a^{2}}\right)$$ (2)

3) Fast Fourier transform (FFT) coefficients: these are the Fourier coefficients of the one-dimensional discrete Fourier transform of real input by a fast Fourier transformation algorithm.

$$A_{k}=\overset{n-1}{\underset{m=0}{\sum }}a_{m}\exp\left\{-2\pi i\frac{mk}{n}\right\},k=0,\ldots ,n-1$$ (3)

4) Mean second derivative central: this is the mean value of a central approximation of the second derivative.

$$A_{d}=\frac{1}{2\left(n-2\right)}\underset{i=1,\ldots ,n-1}{\sum }\frac{1}{2}\left(x_{i+2}-2\cdot x_{i+1}+x_{i}\right)$$ (4)

5) Mean absolute change: this is the mean over the absolute differences between the subsequent time series values.

$$A_{ac}=\frac{1}{n-1}\underset{i=1,\ldots ,n-1}{\sum }\left|x_{i+1}-x_{i}\right|$$ (5)

### 2.4 Machine learning methods and hyperparameter tuning

After the redundant features were removed, the remaining features were input into the LightGBM classifier for blood pressure classification. LightGBM is a new gradient boosting decision tree extension proposed by Microsoft (Ke et al., 2017). The algorithm incorporates exclusive feature bundling (EFB) and gradient-based one-side sampling (GOSS). To optimize the feature values, the algorithm uses the histogram-based algorithm instead of the traditional presorted traversal algorithm. LightGBM offers better model training accuracy and prevention of overfitting than traditional classification algorithms, such as decision trees (Song and Ying, 2015) and random forests (Biau, 2012). To better demonstrate the scientific nature of the experiment, we used a decision tree, AdaBoost, a gradient boosting decision tree (GBDT), random forest, XgBoost, and LightGBM to conduct comparative experiments.

To improve the predictive performance of the LightGBM model and avoid overfitting, a Bayesian optimization library, called Optuna, was employed to effectively adjust the hyperparameters and empirically benchmark its performance. Optuna is a framework created to automate and accelerate hyperparameter optimization experiments (Akiba et al., 2019). It has three core concepts: objective function, single trial, and study. Optuna continually calls for and assesses the objective function for various parameter values to arrive at the best result (Dong et al., 2020; Lacerda et al., 2021). In this study, 1,000 Bayesian optimization trials were used to maximize the accuracy score and 10 LightGBM hyperparameters. Table 1 shows the 10 LightGBM hyperparameters.

**TABLE 1:** Hyperparameter settings of LightGBM used for the Bayesian optimization.

Parameters	Search space
learning_rate	(1e-8, 1.0,'log-uniform')
lambda_l1	(1e-8, 1.0,'log-uniform')
lambda_l2	(1e-6, 1.0,'log-uniform')
bagging_fraction	(0.3, 1.0,'uniform')
feature_fraction	(0.3, 1.0,'uniform')
bagging_freq	(2, 10,'int')
min_child_samples	(1, 50,'int')
max_depth	(2, 20,'int')
num_leaves	(2, 1024,'int’, step = 2)
n_estimators	(100, 4000,'int’, step = 5)

### 2.5 Hypertension classification

**FIGURE 3:**
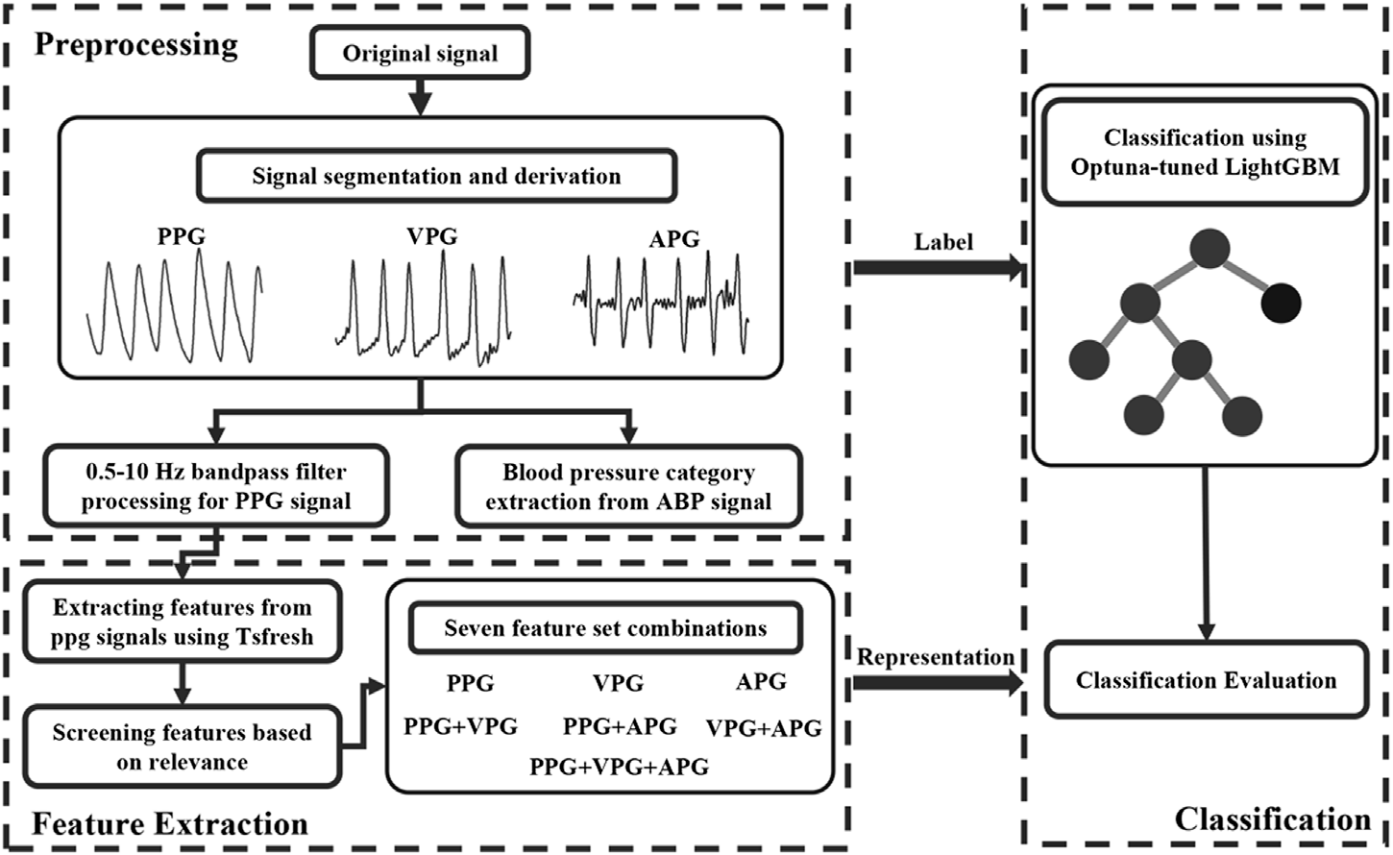
the flow chart of this study. Note: ABP stands for arterial blood pressure, PPG, VPG, and APG denote photoplethysmography, velocity plethysmography, and acceleration plethysmography, respectively.

A recent study (Berstad et al., 2018) showed that multiple binary classifiers resulted in a more robust model than a single network multiclass implementation. In other words, examining several binary classifiers can provide more robust and increased classification accuracy. Here, we focused on formulating the classification problem into a binary classification based on clinical importance. Therefore, we implemented the one-vs-one multiclass and the one-vs-rest multiclass strategies. In the one-vs-one multiclass strategy, it is crucial to differentiate between NT from PHT and NT from HT. With regards to the one-vs-rest multiclass strategy, distinguishing NT + PH from HT is also clinically essential. Consequently, a total of 1,158 NT cases, 950 PHT cases, and 850 HT cases were obtained based on the HT classifications reported by JNC7. Three HT classification trials were established: NT vs. PHT, NT vs. HT, and NT + PHT vs. HT. Seven feature sets were used to classify the different blood pressure categories: one containing only PPG features, one containing only VPG features, one containing only APG features, one containing both PPG and VPG features, one containing both PPG and APG features, one containing both VPG, and APG features, and one containing PPG, VPG, and APG features. We designed these seven feature sets for two purposes. First, we used them to compare the three waveforms, PPG, VPG, and APG, to predict blood pressure levels. Second, we verified whether a feature set containing PPG, VPG, and APG is superior to a single PPG feature set for blood pressure prediction. Figure 3 shows the flow chart for this study.

We used eight assessment methods in this study: specificity (SP), sensitivity (SN), accuracy (ACC), precision (PRE), F1 score, Matthew’s correlation coefficient (MCC), Cohen’s kappa coefficient (Kappa), and area under the receiver operating characteristic (ROC) curve (AUC). These methods are defined as follows:

$$SP=\frac{TN}{TN+FP}$$ (6)

$$SN=\frac{TP}{TP+FN}$$ (7)

$$ACC=\frac{TP+TN}{TP+TN+FT+FN}$$ (8)

$$PRE=\frac{TP}{TP+FP}$$ (9)

$$F1\;score=2\times \frac{TP}{2TP+FP+FN}$$ (10)

$$MCC=\frac{\left(TP\times TN\right)-\left(FP\times FN\right)}{\sqrt{\left(TP+FP\right)\left(TP+FN\right)\left(TN+FP\right)\left(TN+FN\right)}}$$ (11)

$$Kappa=\frac{P_{0}-P_{e}}{1-P_{e}}$$ (12)

TP, FP, TN, and FN stand for true positive, false positive, true negative, and false negative, respectively. $P_{0}$ denotes the level of observed agreement among the raters, and $P_{e}$ indicates the hypothesized likelihood of chance agreement. AUC refers to the area under the ROC curve, representing the sum of the measured classification performance across all possible thresholds.

In this study, a total of 2958 signal segments were obtained and then randomly divided the dataset into 70%, and 30%, of which 70% was the training set with a total of 2070 signal segments and 30% was the testing set with a total of 888 signal segments. The bootstrap method was used based on the recommendation by Xu and Goodacre (2018) who conducted a comparative study of cross-validation, bootstrap, and systematic sampling for estimating the generalization performance of supervised learning. They concluded that most of the resampling methods produce similar correct classification results; therefore, in this study, bootstrap method was implemented instead of the cross-validation method because of the need for parameter optimization using Optuna for LightGBM model tuning. The optimized parameters include the bagging fraction learning control parameter, which indicates the proportion of data for each bootstrap aggregating (bagging); this can improve the robustness of the model.

All signal processing, modeling, and evaluations were performed in PyCharm software (Community version 2020.2.3), developed and distributed by JetBrains (Prague, Czech Republic). Machine learning algorithms were implemented using *Python* 3.8 based on the following packages: LightGBM v3.2.1, Scikit-learn v1.0.1, Optuna v2.10.0, and Tsfresh 0.19.0. The code was executed on a laptop with an Intel i7-6700 as the CPU, 8 GB RAM, and NVIDIA GeForce GTX 960M as the graphics card.

## 3 Results

As traditional classification algorithms, the decision tree and random forest methods have made great contributions to the development of machine learning (Jordan and Mitchell, 2015). To better demonstrate the rigor of this experiment, the performance of these traditional algorithms and LightGBM were compared. Table 2 presents a summary of the performance of the various machine learning models using the PPG signal feature set extracted by Tsfresh, including a decision tree, AdaBoost, GBDT, random forest, XgBoost, and LightGBM, and provides the run times for 100 training runs. The test set included 348 NT cases, 285 PHT cases, and 255 HT cases. The best model performance is marked in bold font. As seen in Table 2, of the six models, the decision tree had the shortest running time but the worst classification performance. The classification performance was slightly better for LightGBM than XgBoost and much better than the other four tested models. The superior performance of the LightGBM model was demonstrated by the fact that it ran in much less time than the XgBoost model.

**TABLE 2:** Classification performance of the proposed machine learning method. Note, NT, PHT, and HT denote normotension, prehypertension, and hypertension, respectively. SP stands for specificity, SN stands for sensitivity, ACC stands for accuracy, PRE stands for precision, MCC stands for Matthew’s correlation coefficient, Kappa stands for Cohen’s kappa coefficient, AUC stands for Area under curve. Values in Bold indicate highest scores achieved for each classification per evaluation metric.

Model	Trail	SP (%)	SN (%)	ACC (%)	PRE (%)	F1 score (%)	AUC (%)	MCC (%)	Kappa (%)	Time (s)
Decision tree	NT vs. PHT	84.44	70.88	72.83	72.89	72.85	72.65	45.22	45.22	**0.23**
	NT vs. HT	80.39	79.60	79.93	80.30	80.02	79.99	59.49	59.34	**0.28**
	(NT + PHT) vs. HT	96.26	65.88	80.41	80.41	80.41	76.07	52.14	52.14	**0.57**
AdaBoost	NT vs. PHT	78.74	71.58	75.51	75.47	75.48	75.16	50.44	50.43	1.64
	NT vs. HT	88.22	75.29	82.75	82.73	82.64	81.76	64.44	64.25	1.59
	(NT + PHT) vs. HT	92.26	60.39	83.11	82.56	82.48	76.33	56.73	56.06	2.47
GBDT	NT vs. PHT	83.62	77.54	80.88	80.86	80.86	80.58	61.32	61.30	7.24
	NT vs. HT	93.10	82.35	88.58	88.62	88.48	87.73	76.50	76.30	6.79
	(NT + PHT) vs. HT	96.37	66.27	87.72	87.75	87.15	81.32	68.85	67.63	11.30
Random forest	NT vs. PHT	84.20	76.14	80.57	80.54	80.52	80.17	60.64	60.59	1.30
	NT vs. HT	93.68	83.14	89.22	89.30	89.15	88.41	77.87	77.67	1.48
	(NT + PHT) vs. HT	96.84	61.57	86.71	86.93	85.90	79.20	66.15	64.28	2.49
XgBoost	NT vs. PHT	85.34	78.95	82.46	82.44	82.44	82.15	64.50	64.48	1.34
	NT vs. HT	93.68	**87.84**	91.21	91.21	91.19	90.76	81.95	81.91	1.09
	(NT + PHT) vs. HT	95.73	**74.90**	89.75	89.63	89.50	**85.32**	74.26	73.84	2.02
LightGBM	NT vs. PHT	**85.92**	**80.70**	**83.57**	**83.55**	**83.55**	**83.31**	**66.76**	**66.75**	0.87
	NT vs. HT	**94.25**	**87.84**	**91.54**	**91.55**	**91.51**	**91.05**	**82.63**	**82.57**	0.81
	(NT + PHT) vs. HT	**96.27**	74.12	**89.98**	**89.92**	**89.68**	85.24	**74.80**	**74.22**	1.13

### 3.1 Hyperparameter tuning

**FIGURE 4:**
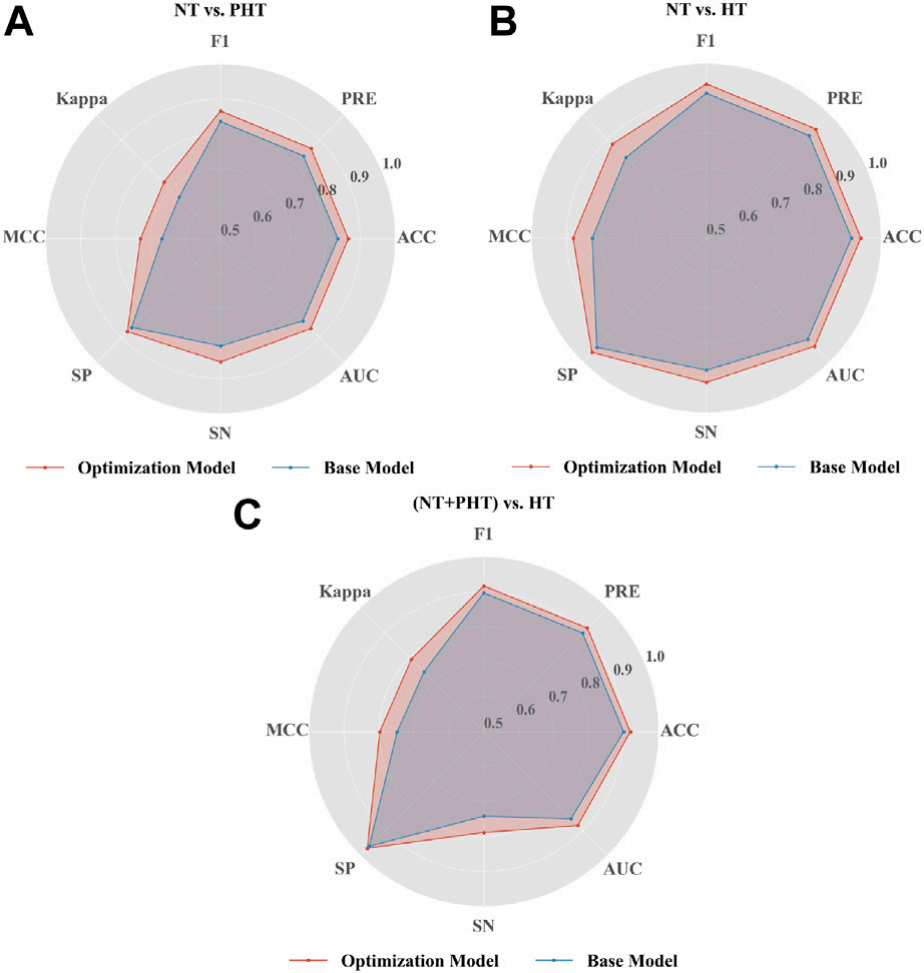
Performance of LightGBM in the classification of hypertension. Radar plot illustrations the performance of base and optimization LightGBM model in **(A)** NT vs. PHT **(B)** NT vs. HT and **(C)** (NT + PHT) vs. HT. Note, NT, PHT, and HT refer to normotension, prehypertension, and hypertension, respectively. SP stands for specificity, SN stands for sensitivity, ACC stands for accuracy, PRE stands for precision, MCC stands for Matthew’s correlation coefficient, Kappa stands for Cohen’s kappa coefficient, AUC stands for Area under curve.

As seen in Figure 4, the optimized LightGBM model with Optuna performed better on the PPG signal feature set extracted by Tsfresh. In the default setting of the LightGBM model, the F1 scores of NT vs. PHT, NT vs. HT, and NT + PHT vs. HT were 0.8355, 0.9151, and 0.8968, respectively. After the Bayesian hyperparameters were modified, the improved LightGBM model performed better in multiple classification tests. The corresponding values for NT vs. PHT, NT vs. HT, and NT + PHT vs. HT were 0.8657, 0.9418, and 0.9170, respectively.

### 3.2 Model performance

To investigate the effects of different feature sets extracted by Tsfresh on blood pressure classification, we used seven different feature sets and trained the Optuna-tuned LightGBM model on each. Table 3 presents a summary of the classification performance of different feature sets using Optuna-tuned LightGBM models. The best performance is marked in bold font. Based on the results presented in Table 3, we reached the following conclusions. First, VPG outperformed PPG and APG in terms of classification performance; the most significant difference in performance improvement was seen between the NT and PHT experiments. Second, the PPG, APG, and VPG datasets outperformed the single PPG dataset, and similar results were obtained in different classification experiments. Third, an increase in the dataset size contributed to an improvement in the performance of blood pressure classification.

**TABLE 3:** Classification performance on different feature sets using Optuna-tuned LightGBM models. Note, NT, PHT, and HT denote normotension, prehypertension, and hypertension, respectively. PPG, VPG, and APG refer to photoplethysmography, velocity plethysmography, and acceleration plethysmography, respectively. SP stands for specificity, SN stands for sensitivity, ACC stands for accuracy, PRE stands for precision, MCC stands for Matthew’s correlation coefficient, Kappa stands for Cohen’s kappa coefficient, AUC stands for Area under curve. Values in Bold indicate highest scores achieved for each classification per evaluation metric.

Feature sets	Trail	SP (%)	SN (%)	ACC (%)	PRE (%)	F1 score (%)	AUC (%)	MCC (%)	Kappa (%)
PPG	NT vs. PHT	87.64	85.26	86.57	86.58	86.57	86.45	72.88	72.88
	NT vs. HT	96.26	91.37	94.20	94.21	94.18	93.82	88.09	88.05
	(NT + PHT) vs. HT	97.16	78.82	91.89	91.89	91.70	87.99	79.75	79.32
VPG	NT vs. PHT	91.09	87.37	89.42	89.41	89.41	89.23	78.60	78.59
	NT vs. HT	96.84	92.16	94.86	94.88	94.85	94.50	89.46	89.42
	(NT + PHT) vs. HT	97.31	79.22	92.12	92.12	91.93	88.27	80.33	79.90
APG	NT vs. PHT	89.94	81.75	86.26	8629	86.21	85.85	72.19	72.09
	NT vs. HT	95.40	94.12	94.86	94.86	94.86	94.76	89.47	89.47
	(NT + PHT) vs. HT	96.21	73.73	89.75	89.68	89.45	84.97	74.22	73.64
PPG and VPG	NT vs. PHT	90.23	**88.77**	89.57	89.58	89.58	89.50	78.95	78.95
	NT vs. HT	97.70	93.33	95.85	95.88	95.84	95.52	91.51	91.47
	(NT + PHT) vs. HT	**97.79**	80.00	92.68	92.73	92.50	88.89	81.78	81.31
PPG and APG	NT vs. PHT	89.94	87.02	88.63	88.62	88.62	88.48	77.01	77.01
	NT vs. HT	97.70	96.08	97.01	97.01	97.01	96.89	93.88	93.88
	(NT + PHT) vs. HT	97.16	80.39	92.34	92.32	92.18	88.77	80.91	90.57
VPG and APG	NT vs. PHT	91.67	85.96	89.10	89.11	89.08	88.82	77.95	77.90
	NT vs. HT	97.41	95.29	96.52	96.52	96.51	96.35	92.85	92.86
	(NT + PHT) vs. HT	97.16	80.00	92.23	92.21	92.06	88.58	80.62	80.26
PPG, VPG and APG	NT vs. PHT	**92.82**	87.02	**90.21**	**90.23**	**90.18**	**89.92**	**80.20**	**80.14**
	NT vs. HT	**98.28**	**96.47**	**97.51**	**97.51**	**97.51**	**97.37**	**94.90**	**94.90**
	(NT + PHT) vs. HT	97.31	**81.96**	**92.91**	**92.89**	**92.77**	**89.64**	**82.34**	**82.06**

### 3.3 Study comparison

In past research (Liang et al., 2018a; Liang et al., 2018b), the dataset used in this study was employed to classify blood pressure using PAT and PPG features extracted from ECG and PPG signals. The results shown in Table 4 denote that the accuracy of the model used in this study is greater than that of the classifier employing PAT and 10 PPG morphological characteristics. Moreover, the method performs better than the Google Net model using continuous wavelet transform. This suggests the potential of the model proposed in this study, which only employed PPG signals and their derivatives, as an alternative to ECG and PPG HT classification methods.

**TABLE 4:** Classification performance of the proposed machine learning method and deep learning method and feature-based methods on the same recordings from the MIMIC-III database. Note, NT, PHT, and HT refer to normotension, prehypertension, and hypertension, respectively. PAT stands for pulse arrival time, CWT stands for continuous wavelet transform, and KNN stands for k-nearest neighbors.

	Trial	Feature	Classifier	F1 (%)
This study (Only PPG)	NT vs. PHT	Tsfresh feature extraction	LightGBM	90.18
	NT vs. HT	Tsfresh feature extraction	LightGBM	97.51
	(NT + PHT) vs. HT	Tsfresh feature extraction	LightGBM	92.77
Based on PAT and PPG features (ECG & PPG) Liang et al. (2018b)	NT vs. PHT	PAT and 10 PPG features	KNN	84.34
	NT vs. HT	PAT and 10 PPG features	KNN	94.84
	(NT + PHT) vs. HT	PAT and 10 PPG features	KNN	88.49
Based on CWT and GoogLeNet (Only PPG) Liang et al. (2018a)	NT vs. PHT	CWT scalogram	GoogLeNet	80.52
	NT vs. HT	CWT scalogram	GoogLeNet	92.55
	(NT + PHT) vs. HT	CWT scalogram	GoogLeNet	82.95

## 4 Discussion

HT is routinely evaluated using blood pressure testing methods. Elderly individuals generally have trouble reading and managing blood pressure cuffs, limiting their use. In contrast, the PPG signal has the advantage of being easy to collect and monitor over time, making it an important tool for noninvasive cardiovascular health screening. However, in a previous study (Chan et al., 2019) based on PPG signals, the researchers had trouble recognizing and extracting feature points because patient age, motion, and respiration all interfere with PPG signals. We have provided a potential solution to these difficulties through using the Tsfresh method, which automatically and robustly extracts features from the original signal and uses the Optuna-tuned LightGBM machine learning model for classification.

LightGBM enables effective parallel training, which can speed up standard GBDT model training 20-fold. It also has reduced memory usage, improved accuracy, and rapid data processing (He et al., 2020). LightGBM has often been used to perform classification and regression tasks (Zeng et al., 2019; Xu et al., 2020). The results shown in Table 2 demonstrate the superior performance of the LightGBM model in terms of training time overhead when using different machine learning models to classify the same dataset.

However, tuning is more difficult for LightGBM than for traditional machine learning techniques, which only need the adjustment of one or two parameters to ensure model correctness and resilience. The grid-search strategy is the most common method for optimizing the 10 LightGBM parameters. However, this method has no pruning operation, resulting in a long search time. Optuna optimization techniques may be used to solve this problem by modifying the hyperparameters (Bergstra et al., 2013). As seen in Figure 3, the baseline LightGBM model could be improved *via* Optuna optimization by over 3.6%, 2.9%, and 2.1% (ACC value), 3.6%, 2.9%, and 2.3% (F-value), 9.2%, 6.6%, and 6.6% (MCC value), and 3.8%, 3.0%, and 3.2% (AUC value) for NT vs. PHT, NT vs. HT, and NT + PHT vs. HT, respectively, thereby verifying the effectiveness of Optuna optimization.

To study the influence of the first- and second-order derivatives of PPG on the classification results, seven different feature sets were used for the classification experiments. The results shown in Table 3 demonstrate that the combined feature set of PPG, VPG, and APG outperformed the single PPG feature set in the blood pressure classification model. VPG denotes the aortic blood flow velocity and APG indicates the change in the velocity of blood flow. Because hypertensive patients have high blood pressure, blood flows more rapidly into the aorta when the aortic valve is open. Additionally, the descending branch of the PPG signal is steeper in hypertensive patients who lack vascular elasticity than in the general population, which is reflected in the APG. Consequently, adding PPG derivative information to the dataset can make blood pressure classification more accurate.

**FIGURE 5:**
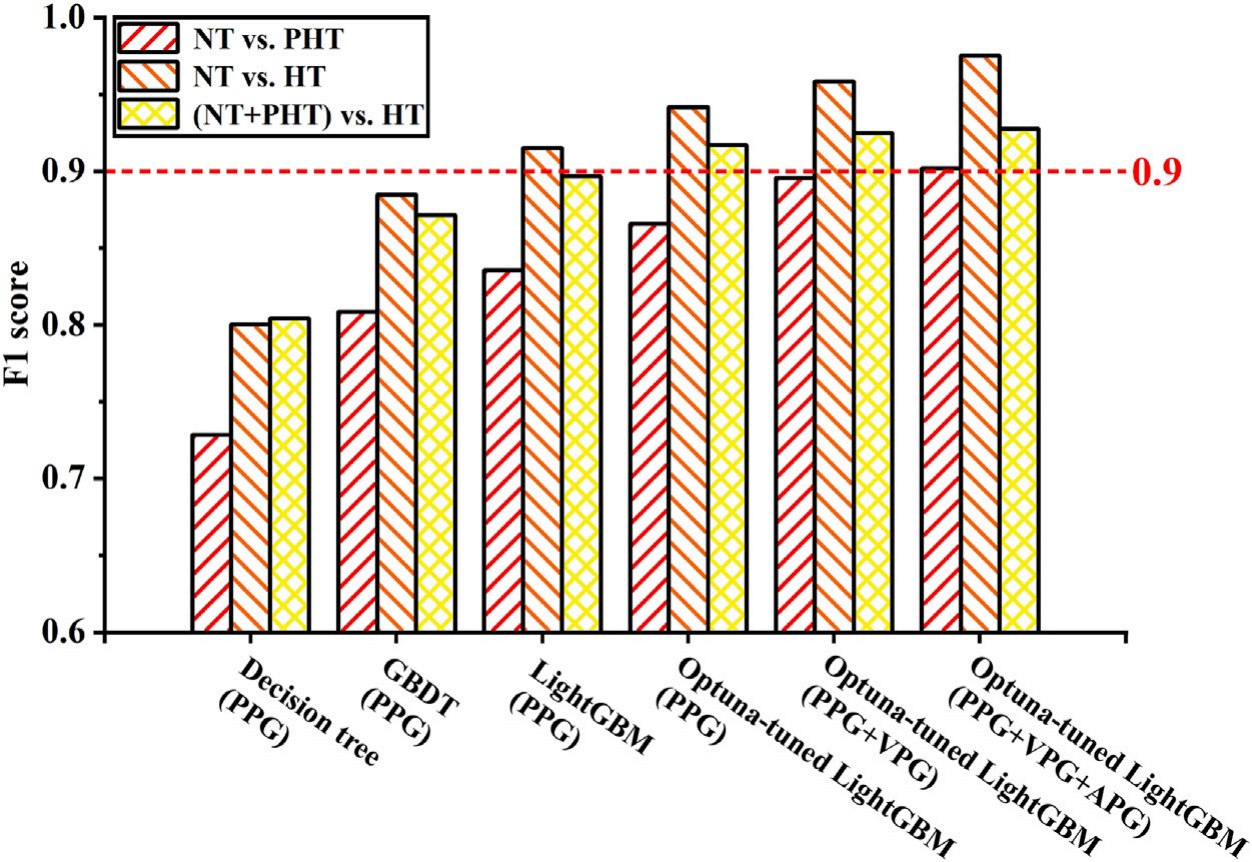
Performance of the model with different feature sets. Note, NT, PHT, and HT represent normotension, prehypertension, and hypertension, respectively. PPG, VPG, and APG denote photoplethysmography, velocity plethysmography, and acceleration plethysmography, respectively.

NT, PHT, and HT are the different stages of blood pressure that the human body exhibits with age or the cause of disease, and also reflect the state of cardiovascular health. Globally, compared with PHT and HT, the number of NT is the largest, from the normotensive population screening to identify PHT and HT samples is of great significance, through one-to-one binary classification research, we can more intuitively observe the actual effect of the proposed scheme in the screening of PHT and HT. Thus, in the machine learning approach using Tsfresh and Optuna-tuned LightGBM, three classification trials for HT were conducted: NT vs. PHT, NT vs. HT, and NT + PHT vs. HT. The classification performance results of the three classification tests are shown in Table 3. The F1 scores of the tests were found to be greater than .85. The combined feature set of PPG, VPG, and APG was associated with the highest F1 scores, with .9018, .9751, and .9277 in different classification experiments, respectively. All the feature sets had F1 scores greater than .9. These findings indicate this method’s potential for detecting HT. Figure 5 shows the performance of the main models used in this study with different feature sets.

It is worth noting that we obtained a total of 1158 NT cases, 950 PHT cases, and 850 HT cases, and the ratios of positive and negative examples for the classification trials were: 1.22 for NT/PHT, 1.36 for NT/HT, and 2.48 for (NT + PHT)/HT. According to García’s study (García et al., 2012), they defined data with positive and negative sample ratios less than 3 as low-imbalance data. They studied the impact of imbalance ratios and classifiers on the performance of several resampling strategies for processing imbalanced datasets and found no significant differences for the low-imbalance data. The data ratios in this paper are all less than 3, which are considered low-imbalance data; therefore, upsampling and downsampling are not needed.

PAT has been examined extensively in novel cuffless blood pressure detection systems (Ding et al., 2017; Zhang et al., 2017). A previous study classified blood pressure using PAT and PPG parameters taken from ECG and PPG signals (Liang et al., 2018b), and the present study used the same dataset. The results presented in Table 4 show that the approach employed in this study has greater accuracy than the method previously utilized. Thus, using the Tsfresh and Optuna-tuned LightGBM method easily yields better performance than extracting PAT and PPG morphological features using the same low-quality signals based on those collected from elderly ICU patients. Therefore, the approach suggested in this research has more practical usefulness and prospective applications.

This research mainly focused on novel blood pressure detection and HT risk categorization, in which extracting features from physiological signals with different qualities has been difficult. It proposed a machine learning-based classification method using Tsfresh for feature extraction and Optuna-tuned LightGBM for HT classification. Experiments were conducted using machine learning techniques, and the results showed that the proposed model has good performance and strong potential for application in the field of wearable cuffless blood pressure measurement.

The method proposed in this study has some advantages and disadvantages. As for the advantages, first, the proposed method does not require the extraction of morphological characteristics. Additionally, there are no special requirements regarding the quality of the PPG signal. Third, this process can be completely automated. Finally, this process does not require a high level of processing power, the procedure is simple, and the processing time is short. These advantages will make it easier to implement the method in wearable cuffless blood pressure management devices. As for the disadvantages, this process is not suitable for the real-time processing of large-scale data and a small dataset was used. One of the next steps is test the performance of the proposed algorithm on a different dataset with bigger sample size with different ethnic groups. Ethnicity plays a major role in creating bias in the PPG signal (Sjoding et al., 2020). The MIMIC database used in this study suffer from ethnicity bias (Sinaki et al., 2022). As the algorithm proposed in the study was trained and tested to differentiate between subjects, the study was designed as an inter-subject stratification approach. The algorithm is expected to perform well in assessing each subject over time if the intra-subject stratification is explored. Although, getting subjects for intra-subject stratification whose blood pressure variation is regularly changing between NT, PH, and PHT is challenging. Such subjects are usually in critical health situations where medication is needed to move them from HT to NT. However, it is an area worth exploring.

## 5 Conclusion

The method proposed in the study discussed in this paper (using Tsfresh and Optuna-tuned LightGBM) increases classification accuracy without requiring the extraction of PPG morphological characteristics or a high-quality PPG signal. Comparison of the results of blood pressure classification trials in various models revealed that our proposed model has higher accuracy than decision tree, AdaBoost, GBDT, random forest, and XgBoost models. Our study also showed that the first- and second-order derivatives of PPG include significant information about blood pressure, allowing PPG, VPG, and APG to be used in place of PAT and PPG for blood pressure prediction. The proposed method automatically diagnoses HT, providing a noninvasive, rapid, and low-cost method for the early detection of HT in low- and middle-income countries.

## Data Availability

The original contributions presented in the study are included in the article/Supplementary Material, further inquiries can be directed to the corresponding authors.
