# Supplementary material for: Blood pressure stratification using photoplethysmography and light gradient boosting machine
Source: Front Physiol. 2023 Feb 20;14:1072273. doi: 10.3389/fphys.2023.1072273 (PMC9986584; doi:10.3389/fphys.2023.1072273)
Supplement: Supplementary file 1 [file Table1.DOCX]

Supplementary Material

# Supplementary Data

## Tsfresh characterization methods

Tsfresh provides 77 time series characterization methods. The following are characterization methods:

1. Absolute energy: this term is the absolute energy of the time series and is the sum of the squared values.

$$\boldsymbol{E=}\sum_{\boldsymbol{i=1,\ldots,n}} \boldsymbol{x}_{\boldsymbol{i}}^{\boldsymbol{2}} \text{ }\text{(1)}$$

2. Absolute maximum: this term is the highest absolute value of the time series.
3. Absolute sum of changes: this term is the sum over the absolute value of consecutive changes in the time series.

$$\boldsymbol{A}_{\boldsymbol{C}}\boldsymbol{=}\sum_{\boldsymbol{i=1,\ldots,n-1}} \boldsymbol{|}\boldsymbol{x}_{\boldsymbol{i+1}}\boldsymbol{-}\boldsymbol{x}_{\boldsymbol{i}}\boldsymbol{|} \text{ (2)}$$

4. Aggregation autocorrelation: this term is the value of an aggregation function over the autocorrelation for different lags.

$$\boldsymbol{R}\left( \boldsymbol{l} \right)\boldsymbol{=}\frac{\boldsymbol{1}}{\left( \boldsymbol{n-l} \right)\boldsymbol{\sigma}^{\boldsymbol{2}}}\sum_{\boldsymbol{t=1}}^{\boldsymbol{n-l}} \left( \boldsymbol{X}_{\boldsymbol{t}}\boldsymbol{-\mu} \right)\left( \boldsymbol{X}_{\boldsymbol{t+l}}\boldsymbol{-\mu} \right) \text{(3)}$$

5. Aggregation linear trend: calculates a linear least-squares regression for values of the time series that were aggregated over chunks versus the sequence from 0 up to the number of chunks minus one.
6. Approximate entropy: implements a vectorized Approximate entropy algorithm.
7. Autoregressive coefficient: Calculates the unconditional maximum likelihood of an autoregressive process.

$$\boldsymbol{X}_{\boldsymbol{t}}\boldsymbol{=}\boldsymbol{\varphi}_{\boldsymbol{0}}\boldsymbol{+}\sum_{\boldsymbol{i=1}}^{\boldsymbol{k}} \boldsymbol{\varphi}_{\boldsymbol{i}}\boldsymbol{X}_{\boldsymbol{t-i}}\boldsymbol{+}\boldsymbol{\varepsilon}_{\boldsymbol{t}} \text{(4)}$$

8. Augmented Dickey-Fuller: the augmented Dickey-Fuller test is a hypothesis test which checks whether a unit root is present in a time series sample. This feature calculator returns the value of the respective test statistic.
9. Autocorrelation: calculates the autocorrelation of the specified lag.

$$\boldsymbol{A}_{\boldsymbol{a}}\boldsymbol{=}\frac{\boldsymbol{1}}{\left( \boldsymbol{n-l} \right)\boldsymbol{\sigma}^{\boldsymbol{2}}}\sum_{\boldsymbol{t=1}}^{\boldsymbol{n-l}} \left( \boldsymbol{X}_{\boldsymbol{t}}\boldsymbol{-\mu} \right)\left( \boldsymbol{X}_{\boldsymbol{t+l}}\boldsymbol{-\mu} \right) \text{ (5)}$$

10. Benford correlation: this term is the correlation from first digit distribution when compared to the Newcomb-Benford’s Law distribution.

$$\boldsymbol{P}\left( \boldsymbol{d} \right)\boldsymbol{=}\mathbf{log}_{\boldsymbol{10}}\left( \boldsymbol{1+}\frac{\boldsymbol{1}}{\boldsymbol{d}} \right) \text{(6)}$$

11. Binned entropy: this term is the max_bins equidistant bins.

$$\boldsymbol{A}_{\boldsymbol{b}}\boldsymbol{=}\sum_{\boldsymbol{k=0}}^{\mathbf{min}\boldsymbol{(max-}\mathbf{bins}\boldsymbol{,}\mathbf{len}\boldsymbol{(x))}} \boldsymbol{p}_{\boldsymbol{k}}\mathbf{log}\left( \boldsymbol{p}_{\boldsymbol{k}} \right)\boldsymbol{\cdot}\mathbf{1}_{\left( \boldsymbol{p}_{\boldsymbol{k}}\boldsymbol{>0} \right)} \text{ (7)}$$

12. C3: uses c3 statistics to measure non linearity in the time series.

$$\boldsymbol{A}_{\boldsymbol{C}\boldsymbol{3}}\boldsymbol{=}\frac{\boldsymbol{1}}{\boldsymbol{n-2}\mathbf{lag}}\sum_{\boldsymbol{i=1}}^{\boldsymbol{n-2}\boldsymbol{lag}} \boldsymbol{x}_{\boldsymbol{i+2}\mathbf{lag}}\boldsymbol{\cdot}\boldsymbol{x}_{\boldsymbol{i+lag}}\boldsymbol{\cdot}\boldsymbol{x}_{\boldsymbol{i}} \text{(8)}$$

13. Change quantiles: calculates the average, absolute value of consecutive changes of the time series inside corridor.
14. Cid ce: this function calculator is an estimate for a time series complexity.

$$\boldsymbol{A}_{\boldsymbol{cc}}\boldsymbol{=}\sqrt{\sum_{\boldsymbol{i=1}}^{\boldsymbol{n-1}} {\boldsymbol{(}\boldsymbol{x}_{\boldsymbol{i}}\boldsymbol{-}\boldsymbol{x}_{\boldsymbol{i-1}}\boldsymbol{)}}^{\boldsymbol{2}}} \text{(9)}$$

15. Count above: this term is the percentage of values in x that are higher than t.
16. Count above mean: this term is the number of values in x that are higher than the mean of x.
17. Count below: this term is the percentage of values in x that are lower than t.
18. Count below mean: this term is the number of values in x that are lower than the mean of x.
19. Continuous wavelet transform (CWT) coefficients: these terms are used to perform a CWT on the Ricker wavelet.

$$\boldsymbol{cwt=}\frac{\boldsymbol{2}}{\sqrt{\boldsymbol{3}\boldsymbol{a}}\boldsymbol{\pi}^{\frac{\boldsymbol{1}}{\boldsymbol{4}}}}\left( \boldsymbol{1-}\frac{\boldsymbol{x}^{\boldsymbol{2}}}{\boldsymbol{a}^{\boldsymbol{2}}} \right)\exp\left( \boldsymbol{-}\frac{\boldsymbol{x}^{\boldsymbol{2}}}{\boldsymbol{2}\boldsymbol{a}^{\boldsymbol{2}}} \right) \text{(10)}$$

20. Energy ratio by chunks: calculates the sum of squares of chunk i out of N chunks expressed as a ratio with the sum of squares over the whole series.
21. Fast Fourier transform (FFT) aggregated: this term is the spectral centroid (mean), variance, skew, and kurtosis of the absolute Fourier transform spectrum.
22. FFT coefficients: these terms are the Fourier coefficients of the one-dimensional discrete Fourier transform of real input by a fast Fourier transformation algorithm.

$$\boldsymbol{A}_{\boldsymbol{k}}\boldsymbol{=}\sum_{\boldsymbol{m=0}}^{\boldsymbol{n}\boldsymbol{-}\boldsymbol{1}} \boldsymbol{a}_{\boldsymbol{m}}\exp\left\{ \boldsymbol{-}\boldsymbol{2}\boldsymbol{\pi i}\frac{\boldsymbol{mk}}{\boldsymbol{n}} \right\}\boldsymbol{,}\quad\boldsymbol{k=0,}\boldsymbol{\ldots}\boldsymbol{,n}\boldsymbol{-}\boldsymbol{1} \text{ (11)}$$

23. First location of maximum: this term is the first location of the maximum value of x. The position is calculated relatively to the length of x.
24. First location of minimum: this term is the first location of the minimal value of x. The position is calculated relatively to the length of x.
25. Fourier entropy: calculate the binned entropy of the power spectral density of the time series.
26. Friedrich coefficients: coefficients of polynomial h(x), which has been fitted to the deterministic dynamics of Langevin model.
27. Has duplicate: checks if any value in x occurs more than once.
28. Has duplicate max: checks if the maximum value of x is observed more than once.
29. Has duplicate min: checks if the minimal value of x is observed more than once.
30. Index mass quantile: calculates the relative index i of time series x where q% of the mass of x lies left of i.
31. Kurtosis: this term is the kurtosis of x (calculated with the adjusted Fisher-Pearson standardized moment coefficient G2).
32. Large standard deviation: this term is the Boolean variable denoting if the standard dev of x is higher than ‘r’ times the range (difference between max and min of x).

$$\mathbf{std}\left( \boldsymbol{x} \right)\boldsymbol{>r*}\left( \boldsymbol{max}\left( \boldsymbol{X} \right)\boldsymbol{-min}\left( \boldsymbol{X} \right) \right) \text{(13)}$$

33. Last location of maximum: this term is the relative last location of the maximum value of the time series x.
34. Last location of minimum: this term is the last location of the minimal value of the time series x.
35. Lempel-Ziv complexity: calculate a complexity estimate based on the Lempel-Ziv compression algorithm.
36. Length: this term is the length of the time series.
37. Linear trend: calculate a linear least-squares regression for the values of the time series versus the sequence from 0 to length of the time series minus one (the output format is pandas.Series).
38. Linear trend timewise: Calculate a linear least-squares regression for the values of the time series versus the sequence from 0 to length of the time series minus one (the output format is list).
39. Longest strike above mean: this term is the length of the longest consecutive subsequence in x that is bigger than the mean of x.
40. Longest strike below mean: this term is the length of the longest consecutive subsequence in x that is smaller than the mean of x.
41. Matrix Profile: calculates the 1-D Matrix Profile and returns Tukey’s Five Number Set plus the mean of that Matrix Profile.
42. Max langevin fixed point: this term is the largest fixed point of dynamics.
43. Maximum: this term is the highest value of the time series x.
44. Mean: this term is the mean of the time series x.
45. Mean absolute change: this term is the mean over the absolute differences between subsequent time series values.

$$\boldsymbol{A}_{\boldsymbol{ac}}\boldsymbol{=}\frac{\boldsymbol{1}}{\boldsymbol{n-1}}\sum_{\boldsymbol{i=1,\ldots,n-1}} \left| \boldsymbol{x}_{\boldsymbol{i+1}}\boldsymbol{-}\boldsymbol{x}_{\boldsymbol{i}} \right| \text{(15)}$$

46. Mean change: this term is the mean over the differences between subsequent time series values.

$$\boldsymbol{A}_{\boldsymbol{mc}}\boldsymbol{=}\frac{\boldsymbol{1}}{\boldsymbol{n-1}}\sum_{\boldsymbol{i=1,\ldots,n-1}} \boldsymbol{x}_{\boldsymbol{i+1}}\boldsymbol{-}\boldsymbol{x}_{\boldsymbol{i}} \text{ (16)}$$

47. Mean n absolute max: this term is the arithmetic mean of the n absolute maximum values of the time series.
48. Mean second derivative central: this term is the mean value of a central approximation of the second derivative.

$$\boldsymbol{A}_{\boldsymbol{d}}\boldsymbol{=}\frac{\boldsymbol{1}}{\boldsymbol{2(n}\boldsymbol{-}\boldsymbol{2)}}\sum_{\boldsymbol{i=1,}\boldsymbol{\ldots}\boldsymbol{,n}\boldsymbol{-}\boldsymbol{1}} \frac{\boldsymbol{1}}{\boldsymbol{2}}\left( \boldsymbol{x}_{\boldsymbol{i+2}}\boldsymbol{-}\boldsymbol{2}\boldsymbol{\cdot}\boldsymbol{x}_{\boldsymbol{i+1}}\boldsymbol{+}\boldsymbol{x}_{\boldsymbol{i}} \right) \text{(17)}$$

49. Median: this term is the median of the time series x.
50. Minimum: this term is the lowest value of the time series x.
51. Number crossing m: calculates the number of crossings of x on m. A crossing is defined as two sequential values where the first value is lower than m and the next is greater, or vice-versa.
52. Number CWT peaks: this term is the number of different peaks in the time series x.
53. Number peaks: calculates the number of peaks of at least support n in the time series x. A peak of support n is defined as a subsequence of x where a value occurs, which is bigger than its n neighbours to the left and to the right.
54. Partial autocorrelation: calculates the value of the partial autocorrelation function at the given lag.

$$\boldsymbol{\alpha}_{\boldsymbol{k}}\boldsymbol{=}\frac{\mathbf{Cov} \boldsymbol{(}\boldsymbol{x}_{\boldsymbol{t}}\boldsymbol{,}\boldsymbol{x}_{\boldsymbol{t-k}}\boldsymbol{\mid}\boldsymbol{x}_{\boldsymbol{t-1}}\boldsymbol{,\ldots,}\boldsymbol{x}_{\boldsymbol{t-k+1}}\boldsymbol{)}}{\sqrt{\mathbf{Var}\boldsymbol{(}\boldsymbol{x}_{\boldsymbol{t}}\boldsymbol{\mid}\boldsymbol{x}_{\boldsymbol{t-1}}\boldsymbol{,\ldots,}\boldsymbol{x}_{\boldsymbol{t-k+1}}\boldsymbol{)}\mathbf{Var}\boldsymbol{(}\boldsymbol{x}_{\boldsymbol{t-k}}\boldsymbol{\mid}\boldsymbol{x}_{\boldsymbol{t-1}}\boldsymbol{,\ldots,}\boldsymbol{x}_{\boldsymbol{t-k+1}}\boldsymbol{)}}} \text{(18)}$$

55. Percentage of reoccurring datapoints to all datapoints: this term is the percentage of non-unique data points.
56. Percentage of reoccurring values to all values: this term is the percentage of values that are present in the time series more than once.
57. Permutation entropy: calculate the permutation entropy.
58. Quantile: calculates the q quantile of x. This is the value of x greater than q% of the ordered values from x.
59. Query similarity count: this feature calculator accepts an input query subsequence parameter, compares the query (under z-normalized Euclidean distance) to all subsequences within the time series, and returns a count of the number of times the query was found in the time series (within some predefined maximum distance threshold).
60. Range count: count observed values within the interval [min, max).
61. Ratio beyond r sigma: this term is the ratio of values that are more than r * std(x) away from the mean of x.
62. Ratio value number to time series length: this term is a factor which is 1 if all values in the time series occur only once, and below one if this is not the case.
63. Root mean square: this term is the root mean square of the time series.
64. Sample entropy: calculate and return sample entropy of x.
65. Set property: this method returns a decorator that sets the property key of the function to value.
66. Skewness: this term is the sample skewness of x (calculated with the adjusted Fisher-Pearson standardized moment coefficient G1).
67. Spkt welch density: this feature calculator estimates the cross power spectral density of the time series x at different frequencies.
68. Standard deviation: this term is the standard deviation of x.
69. Sum of reoccurring data points: this term is the sum of all data points, that are present in the time series more than once.
70. Sum of reoccurring values: this term is the sum of all values, that are present in the time series more than once.
71. Sum values: calculates the sum over the time series values.
72. Symmetry looking: this term is the Boolean variable denoting if the distribution of x looks symmetric.

$$\boldsymbol{|}\mathbf{mean}\boldsymbol{(X)-}\mathbf{median}\boldsymbol{(X)|<r*}\left( \boldsymbol{max}\left( \boldsymbol{X} \right)\boldsymbol{-min}\left( \boldsymbol{X} \right) \right) \text{(19)}$$

73. Time reversal asymmetry statistic: this term is the time reversal asymmetry statistic.

$$\boldsymbol{A}_{\boldsymbol{as}}\boldsymbol{=}\frac{\boldsymbol{1}}{\boldsymbol{n-2}\mathbf{lag}}\sum_{\boldsymbol{i=1}}^{\boldsymbol{n-2}\boldsymbol{lag}} \boldsymbol{x}_{\boldsymbol{i+2\cdot lag}}^{\boldsymbol{2}}\boldsymbol{\cdot}\boldsymbol{x}_{\boldsymbol{i+lag}}\boldsymbol{-}\boldsymbol{x}_{\boldsymbol{i+lag}}\boldsymbol{\cdot}\boldsymbol{x}_{\boldsymbol{i}}^{\boldsymbol{2}} \text{ (20)}$$

74. Value count: count occurrences of value in time series x.
75. Variance: this term is the variance of the time series x.
76. Variance larger than standard deviation: this term is the Boolean variable denoting if the variance of x is greater than its standard deviation.
77. Variation coefficient: this term is the variation coefficient (standard error / mean, give relative value of variation around mean) of x.

# Supplementary Tables

## PPG signal segment characteristics

In this study, Tsfresh method was used to extract the features of PPG timing subsegment, and then the obtained features were screened, and a total of 189 features were obtained. Table 1 shows the final 189 features obtained.

**Supplementary Table 1.** A total of 189 features were obtained for each PPG timing subsegment. Note, CWT stands for Continuous wavelet transform, FFT stands for Fast Fourier transform.

| **characterization methods** | **features** |
| --- | --- |
| Absolute energy | absolute_sum_of_changes |
| Aggregation autocorrelation | agg_autocorrelation__f_agg_"mean"__maxlag_40  agg_autocorrelation__f_agg_"median"__maxlag_40  agg_autocorrelation__f_agg_"var"__maxlag_40 |
| Aggregation linear trend | agg_linear_trend__attr_"intercept"__chunk_len_10__f_agg_"var"  agg_linear_trend__attr_"intercept"__chunk_len_5__f_agg_"var"  agg_linear_trend__attr_"intercept"__chunk_len_50__f_agg_"max"  agg_linear_trend__attr_"intercept"__chunk_len_50__f_agg_"var"  agg_linear_trend__attr_"stderr"__chunk_len_10__f_agg_"max"  agg_linear_trend__attr_"stderr"__chunk_len_10__f_agg_"mean"  agg_linear_trend__attr_"stderr"__chunk_len_10__f_agg_"min"  agg_linear_trend__attr_"stderr"__chunk_len_5__f_agg_"max"  agg_linear_trend__attr_"stderr"__chunk_len_5__f_agg_"min"  agg_linear_trend__attr_"stderr"__chunk_len_50__f_agg_"min" |
| Approximate entropy | approximate_entropy__m_2__r_0.3  approximate_entropy__m_2__r_0.5  approximate_entropy__m_2__r_0.7  approximate_entropy__m_2__r_0.9 |
| Autoregressive coefficient | ar_coefficient__coeff_1__k_10  ar_coefficient__coeff_2__k_10  ar_coefficient__coeff_3__k_10  ar_coefficient__coeff_4__k_10  ar_coefficient__coeff_5__k_10  ar_coefficient__coeff_6__k_10  ar_coefficient__coeff_7__k_10  ar_coefficient__coeff_8__k_10 |
| Autocorrelation | autocorrelation__lag_1  autocorrelation__lag_2  autocorrelation__lag_3  autocorrelation__lag_4  autocorrelation__lag_5  autocorrelation__lag_6  autocorrelation__lag_7  autocorrelation__lag_8  autocorrelation__lag_9 |
| Benford correlation | benford_correlation |
| C3 | c3__lag_1  c3__lag_2  c3__lag_3 |
| Change quantiles | change_quantiles__f_agg_"mean"__isabs_False__qh_0.8__ql_0.6  change_quantiles__f_agg_"mean"__isabs_False__qh_1.0__ql_0.8  change_quantiles__f_agg_"mean"__isabs_True__qh_0.4__ql_0.0  change_quantiles__f_agg_"mean"__isabs_True__qh_0.6__ql_0.4  change_quantiles__f_agg_"mean"__isabs_True__qh_0.8__ql_0.4  change_quantiles__f_agg_"mean"__isabs_True__qh_0.8__ql_0.6  change_quantiles__f_agg_"mean"__isabs_True__qh_1.0__ql_0.0  change_quantiles__f_agg_"mean"__isabs_True__qh_1.0__ql_0.2  change_quantiles__f_agg_"mean"__isabs_True__qh_1.0__ql_0.4  change_quantiles__f_agg_"mean"__isabs_True__qh_1.0__ql_0.6  change_quantiles__f_agg_"mean"__isabs_True__qh_1.0__ql_0.8  change_quantiles__f_agg_"var"__isabs_False__qh_0.2__ql_0.0 change_quantiles__f_agg_"var"__isabs_False__qh_0.4__ql_0.0  change_quantiles__f_agg_"var"__isabs_False__qh_0.4__ql_0.2  change_quantiles__f_agg_"var"__isabs_False__qh_0.6__ql_0.4  change_quantiles__f_agg_"var"__isabs_False__qh_0.8__ql_0.6  change_quantiles__f_agg_"var"__isabs_False__qh_1.0__ql_0.0  change_quantiles__f_agg_"var"__isabs_False__qh_1.0__ql_0.2  change_quantiles__f_agg_"var"__isabs_False__qh_1.0__ql_0.4  change_quantiles__f_agg_"var"__isabs_False__qh_1.0__ql_0.6  change_quantiles__f_agg_"var"__isabs_False__qh_1.0__ql_0.8  change_quantiles__f_agg_"var"__isabs_True__qh_0.2__ql_0.0  change_quantiles__f_agg_"var"__isabs_True__qh_0.4__ql_0.0  change_quantiles__f_agg_"var"__isabs_True__qh_0.4__ql_0.2  change_quantiles__f_agg_"var"__isabs_True__qh_0.6__ql_0.0  change_quantiles__f_agg_"var"__isabs_True__qh_0.6__ql_0.4  change_quantiles__f_agg_"var"__isabs_True__qh_0.8__ql_0.0  change_quantiles__f_agg_"var"__isabs_True__qh_0.8__ql_0.2  change_quantiles__f_agg_"var"__isabs_True__qh_0.8__ql_0.4  change_quantiles__f_agg_"var"__isabs_True__qh_0.8__ql_0.6  change_quantiles__f_agg_"var"__isabs_True__qh_1.0__ql_0.8 |
| Cid ce | cid_ce__normalize_False  cid_ce__normalize_True |
| Count above | count_above__t_0 |
| Count above mean | count_above_mean |
| Count below | count_below__t_0 |
| Count below mean | count_below_mean |
| FFT aggregated | fft_aggregated__aggtype_"centroid"  fft_aggregated__aggtype_"kurtosis"  fft_aggregated__aggtype_"skew"  fft_aggregated__aggtype_"variance" |
| FFT coefficients | fft_coefficient__attr_"abs"__coeff_5  fft_coefficient__attr_"abs"__coeff_6  fft_coefficient__attr_"abs"__coeff_7  fft_coefficient__attr_"abs"__coeff_9  fft_coefficient__attr_"abs"__coeff_10  fft_coefficient__attr_"abs"__coeff_12  fft_coefficient__attr_"abs"__coeff_14  fft_coefficient__attr_"abs"__coeff_18  fft_coefficient__attr_"abs"__coeff_21  fft_coefficient__attr_"abs"__coeff_25  fft_coefficient__attr_"abs"__coeff_26  fft_coefficient__attr_"abs"__coeff_28  fft_coefficient__attr_"abs"__coeff_29  fft_coefficient__attr_"abs"__coeff_30  fft_coefficient__attr_"abs"__coeff_31  fft_coefficient__attr_"abs"__coeff_32  fft_coefficient__attr_"abs"__coeff_33  fft_coefficient__attr_"abs"__coeff_34  fft_coefficient__attr_"abs"__coeff_35  fft_coefficient__attr_"abs"__coeff_36  fft_coefficient__attr_"abs"__coeff_37  fft_coefficient__attr_"abs"__coeff_38  fft_coefficient__attr_"abs"__coeff_39  fft_coefficient__attr_"abs"__coeff_40  fft_coefficient__attr_"abs"__coeff_44  fft_coefficient__attr_"abs"__coeff_50  fft_coefficient__attr_"abs"__coeff_53  fft_coefficient__attr_"abs"__coeff_60  fft_coefficient__attr_"abs"__coeff_61  fft_coefficient__attr_"abs"__coeff_62  fft_coefficient__attr_"abs"__coeff_63  fft_coefficient__attr_"abs"__coeff_64  fft_coefficient__attr_"abs"__coeff_65  fft_coefficient__attr_"abs"__coeff_66  fft_coefficient__attr_"abs"__coeff_67  fft_coefficient__attr_"abs"__coeff_68  fft_coefficient__attr_"abs"__coeff_69  fft_coefficient__attr_"abs"__coeff_70  fft_coefficient__attr_"abs"__coeff_71  fft_coefficient__attr_"abs"__coeff_72  fft_coefficient__attr_"abs"__coeff_73  fft_coefficient__attr_"abs"__coeff_74  fft_coefficient__attr_"abs"__coeff_75  fft_coefficient__attr_"abs"__coeff_76  fft_coefficient__attr_"abs"__coeff_77  fft_coefficient__attr_"abs"__coeff_78  fft_coefficient__attr_"abs"__coeff_79  fft_coefficient__attr_"abs"__coeff_80  fft_coefficient__attr_"abs"__coeff_81  fft_coefficient__attr_"abs"__coeff_82  fft_coefficient__attr_"abs"__coeff_83  fft_coefficient__attr_"abs"__coeff_84  fft_coefficient__attr_"abs"__coeff_85  fft_coefficient__attr_"abs"__coeff_86  fft_coefficient__attr_"abs"__coeff_87  fft_coefficient__attr_"abs"__coeff_88  fft_coefficient__attr_"abs"__coeff_89  fft_coefficient__attr_"abs"__coeff_90  fft_coefficient__attr_"abs"__coeff_91  fft_coefficient__attr_"abs"__coeff_92  fft_coefficient__attr_"abs"__coeff_93  fft_coefficient__attr_"abs"__coeff_94  fft_coefficient__attr_"abs"__coeff_95  fft_coefficient__attr_"abs"__coeff_96  fft_coefficient__attr_"abs"__coeff_97  fft_coefficient__attr_"abs"__coeff_98  fft_coefficient__attr_"abs"__coeff_99 |
| Fourier entropy | fourier_entropy__bins_10  fourier_entropy__bins_100 |
| Friedrich coefficients | friedrich_coefficients__coeff_1__m_3__r_30  friedrich_coefficients__coeff_2__m_3__r_30  friedrich_coefficients__coeff_3__m_3__r_30 |
| Lempel-Ziv complexity | lempel_ziv_complexity__bins_10  lempel_ziv_complexity__bins_5 |
| Longest strike above mean | longest_strike_above_mean |
| Longest strike below mean | longest_strike_below_mean |
| Maximum | maximum |
| Mean absolute change | mean_abs_change |
| Median | median |
| Minimum | minimum |
| Number crossing m | number_crossing_m__m_0  number_crossing_m__m_1 |
| Number CWT peaks | number_cwt_peaks__n_1  number_cwt_peaks__n_5 |
| Number peaks | number_peaks__n_1  number_peaks__n_50 |
| Partial autocorrelation | partial_autocorrelation__lag_1  partial_autocorrelation__lag_2  partial_autocorrelation__lag_3  partial_autocorrelation__lag_4  partial_autocorrelation__lag_5  partial_autocorrelation__lag_6  partial_autocorrelation__lag_7  partial_autocorrelation__lag_8 |
| Permutation entropy | permutation_entropy__dimension_5__tau_1  permutation_entropy__dimension_6__tau_1  permutation_entropy__dimension_7__tau_1 |
| Quantile | quantile__q_0.1  quantile__q_0.3  quantile__q_0.4  quantile__q_0.8  quantile__q_0.9 |
| Range count | range_count__max_1000000000000.0__min_0 |
| Ratio beyond r sigma | ratio_beyond_r_sigma__r_0.5 |
| Skewness | skewness |
| Spkt welch density | spkt_welch_density__coeff_2  spkt_welch_density__coeff_5  spkt_welch_density__coeff_8 |
| Symmetry looking | symmetry_looking__r_0.1 |

## VPG signal segment characteristics

In this study, VPG timing subsegment used the same feature processing method as PPG and finally got 200 features. Table 2 shows the final 200 features obtained.

**Supplementary Table 2.** A total of 200 features were obtained for each VPG timing subsegment. Note, CWT stands for Continuous wavelet transform, FFT stands for Fast Fourier transform.

| **characterization methods** | **features** |
| --- | --- |
| Absolute energy | absolute_sum_of_changes |
| Aggregation autocorrelation | agg_autocorrelation__f_agg_"mean"__maxlag_40  agg_autocorrelation__f_agg_"median"__maxlag_40  agg_autocorrelation__f_agg_"var"__maxlag_40 |
| Aggregation linear trend | agg_linear_trend__attr_"intercept"__chunk_len_10__f_agg_"var"  agg_linear_trend__attr_"intercept"__chunk_len_5__f_agg_"var"  agg_linear_trend__attr_"stderr"__chunk_len_10__f_agg_"max"  agg_linear_trend__attr_"stderr"__chunk_len_10__f_agg_"mean"  agg_linear_trend__attr_"stderr"__chunk_len_10__f_agg_"var"  agg_linear_trend__attr_"stderr"__chunk_len_5__f_agg_"max"  agg_linear_trend__attr_"stderr"__chunk_len_5__f_agg_"mean"  agg_linear_trend__attr_"stderr"__chunk_len_5__f_agg_"min"  agg_linear_trend__attr_"stderr"__chunk_len_5__f_agg_"var"  agg_linear_trend__attr_"stderr"__chunk_len_50__f_agg_"max"  agg_linear_trend__attr_"stderr"__chunk_len_50__f_agg_"min"  agg_linear_trend__attr_"stderr"__chunk_len_50__f_agg_"var" |
| Approximate entropy | approximate_entropy__m_2__r_0.1  approximate_entropy__m_2__r_0.7  approximate_entropy__m_2__r_0.9 |
| Autoregressive coefficient | ar_coefficient__coeff_1__k_10  ar_coefficient__coeff_2__k_10  ar_coefficient__coeff_3__k_10  ar_coefficient__coeff_4__k_10  ar_coefficient__coeff_5__k_10  ar_coefficient__coeff_6__k_10  ar_coefficient__coeff_7__k_10  ar_coefficient__coeff_8__k_10  ar_coefficient__coeff_9__k_10  ar_coefficient__coeff_10__k_10 |
| Augmented Dickey-Fuller | augmented_dickey_fuller__attr_"pvalue"__autolag_"AIC"  augmented_dickey_fuller__attr_"teststat"__autolag_"AIC" |
| Autocorrelation | autocorrelation__lag_1  autocorrelation__lag_2  autocorrelation__lag_3  autocorrelation__lag_4  autocorrelation__lag_5  autocorrelation__lag_6  autocorrelation__lag_7  autocorrelation__lag_8  autocorrelation__lag_9 |
| Binned entropy | binned_entropy__max_bins_10 |
| C3 | c3__lag_1  c3__lag_2 |
| Change quantiles | change_quantiles__f_agg_"mean"__isabs_False__qh_0.2__ql_0.0  change_quantiles__f_agg_"mean"__isabs_False__qh_0.4__ql_0.0  change_quantiles__f_agg_"mean"__isabs_False__qh_0.4__ql_0.2  change_quantiles__f_agg_"mean"__isabs_False__qh_0.6__ql_0.0  change_quantiles__f_agg_"mean"__isabs_False__qh_0.6__ql_0.2  change_quantiles__f_agg_"mean"__isabs_False__qh_0.6__ql_0.4  change_quantiles__f_agg_"mean"__isabs_False__qh_0.8__ql_0.0  change_quantiles__f_agg_"mean"__isabs_False__qh_0.8__ql_0.2  change_quantiles__f_agg_"mean"__isabs_False__qh_0.8__ql_0.4  change_quantiles__f_agg_"mean"__isabs_False__qh_0.8__ql_0.6  change_quantiles__f_agg_"mean"__isabs_False__qh_1.0__ql_0.4  change_quantiles__f_agg_"mean"__isabs_False__qh_1.0__ql_0.6  change_quantiles__f_agg_"mean"__isabs_False__qh_1.0__ql_0.8  change_quantiles__f_agg_"mean"__isabs_True__qh_0.2__ql_0.0  change_quantiles__f_agg_"mean"__isabs_True__qh_0.4__ql_0.0  change_quantiles__f_agg_"mean"__isabs_True__qh_0.4__ql_0.2  change_quantiles__f_agg_"mean"__isabs_True__qh_0.6__ql_0.0  change_quantiles__f_agg_"mean"__isabs_True__qh_0.6__ql_0.2  change_quantiles__f_agg_"mean"__isabs_True__qh_0.8__ql_0.2  change_quantiles__f_agg_"mean"__isabs_True__qh_0.8__ql_0.4  change_quantiles__f_agg_"mean"__isabs_True__qh_0.8__ql_0.6  change_quantiles__f_agg_"mean"__isabs_True__qh_1.0__ql_0.0  change_quantiles__f_agg_"mean"__isabs_True__qh_1.0__ql_0.8  change_quantiles__f_agg_"var"__isabs_False__qh_0.2__ql_0.0  change_quantiles__f_agg_"var"__isabs_False__qh_0.4__ql_0.0  change_quantiles__f_agg_"var"__isabs_False__qh_0.4__ql_0.2  change_quantiles__f_agg_"var"__isabs_False__qh_0.6__ql_0.0  change_quantiles__f_agg_"var"__isabs_False__qh_0.6__ql_0.2  change_quantiles__f_agg_"var"__isabs_False__qh_0.8__ql_0.2  change_quantiles__f_agg_"var"__isabs_False__qh_0.8__ql_0.4  change_quantiles__f_agg_"var"__isabs_False__qh_0.8__ql_0.6  change_quantiles__f_agg_"var"__isabs_False__qh_1.0__ql_0.0  change_quantiles__f_agg_"var"__isabs_False__qh_1.0__ql_0.2  change_quantiles__f_agg_"var"__isabs_False__qh_1.0__ql_0.4  change_quantiles__f_agg_"var"__isabs_False__qh_1.0__ql_0.6  change_quantiles__f_agg_"var"__isabs_False__qh_1.0__ql_0.8  change_quantiles__f_agg_"var"__isabs_True__qh_0.2__ql_0.0  change_quantiles__f_agg_"var"__isabs_True__qh_0.4__ql_0.0  change_quantiles__f_agg_"var"__isabs_True__qh_0.4__ql_0.2  change_quantiles__f_agg_"var"__isabs_True__qh_0.6__ql_0.0  change_quantiles__f_agg_"var"__isabs_True__qh_0.6__ql_0.2  change_quantiles__f_agg_"var"__isabs_True__qh_0.8__ql_0.0  change_quantiles__f_agg_"var"__isabs_True__qh_0.8__ql_0.2  change_quantiles__f_agg_"var"__isabs_True__qh_0.8__ql_0.4  change_quantiles__f_agg_"var"__isabs_True__qh_0.8__ql_0.6  change_quantiles__f_agg_"var"__isabs_True__qh_1.0__ql_0.0  change_quantiles__f_agg_"var"__isabs_True__qh_1.0__ql_0.2  change_quantiles__f_agg_"var"__isabs_True__qh_1.0__ql_0.4  change_quantiles__f_agg_"var"__isabs_True__qh_1.0__ql_0.6  change_quantiles__f_agg_"var"__isabs_True__qh_1.0__ql_0.8 |
| Cid ce | cid_ce__normalize_False  cid_ce__normalize_True |
| FFT coefficients | fft_coefficient__attr_"abs"__coeff_5  fft_coefficient__attr_"abs"__coeff_6  fft_coefficient__attr_"abs"__coeff_7  fft_coefficient__attr_"abs"__coeff_9  fft_coefficient__attr_"abs"__coeff_10  fft_coefficient__attr_"abs"__coeff_13  fft_coefficient__attr_"abs"__coeff_14  fft_coefficient__attr_"abs"__coeff_18  fft_coefficient__attr_"abs"__coeff_21  fft_coefficient__attr_"abs"__coeff_23  fft_coefficient__attr_"abs"__coeff_25  fft_coefficient__attr_"abs"__coeff_26  fft_coefficient__attr_"abs"__coeff_27  fft_coefficient__attr_"abs"__coeff_28  fft_coefficient__attr_"abs"__coeff_29  fft_coefficient__attr_"abs"__coeff_30  fft_coefficient__attr_"abs"__coeff_31  fft_coefficient__attr_"abs"__coeff_32  fft_coefficient__attr_"abs"__coeff_33  fft_coefficient__attr_"abs"__coeff_34  fft_coefficient__attr_"abs"__coeff_35  fft_coefficient__attr_"abs"__coeff_36  fft_coefficient__attr_"abs"__coeff_37  fft_coefficient__attr_"abs"__coeff_38  fft_coefficient__attr_"abs"__coeff_39  fft_coefficient__attr_"abs"__coeff_40  fft_coefficient__attr_"abs"__coeff_42  fft_coefficient__attr_"abs"__coeff_43  fft_coefficient__attr_"abs"__coeff_44  fft_coefficient__attr_"abs"__coeff_45  fft_coefficient__attr_"abs"__coeff_46  fft_coefficient__attr_"abs"__coeff_47  fft_coefficient__attr_"abs"__coeff_48  fft_coefficient__attr_"abs"__coeff_49  fft_coefficient__attr_"abs"__coeff_50  fft_coefficient__attr_"abs"__coeff_51  fft_coefficient__attr_"abs"__coeff_52  fft_coefficient__attr_"abs"__coeff_53  fft_coefficient__attr_"abs"__coeff_54  fft_coefficient__attr_"abs"__coeff_55  fft_coefficient__attr_"abs"__coeff_56  fft_coefficient__attr_"abs"__coeff_58  fft_coefficient__attr_"abs"__coeff_59  fft_coefficient__attr_"abs"__coeff_61 |
| Fourier entropy | fourier_entropy__bins_100  fourier_entropy__bins_2  fourier_entropy__bins_3 |
| Friedrich coefficients | friedrich_coefficients__coeff_1__m_3__r_30  friedrich_coefficients__coeff_2__m_3__r_30  friedrich_coefficients__coeff_3__m_3__r_30 |
| Kurtosis | kurtosis |
| Large standard deviation | large_standard_deviation__r_0.15000000000000002  large_standard_deviation__r_0.2  large_standard_deviation__r_0.25 |
| Lempel-Ziv complexity | lempel_ziv_complexity__bins_10  lempel_ziv_complexity__bins_100  lempel_ziv_complexity__bins_3  lempel_ziv_complexity__bins_5 |
| Longest strike below mean | longest_strike_below_mean |
| Max langevin fixed point | max_langevin_fixed_point__m_3__r_30 |
| Maximum | maximum |
| Mean absolute change | mean_abs_change |
| Minimum | minimum |
| Number crossing m | number_crossing_m__m_0  number_crossing_m__m_1 |
| Number CWT peaks | number_cwt_peaks__n_1  number_cwt_peaks__n_5 |
| Number peaks | number_peaks__n_1  number_peaks__n_10  number_peaks__n_3  number_peaks__n_5  number_peaks__n_50 |
| Partial autocorrelation | partial_autocorrelation__lag_1  partial_autocorrelation__lag_2  partial_autocorrelation__lag_3  partial_autocorrelation__lag_4  partial_autocorrelation__lag_5  partial_autocorrelation__lag_6  partial_autocorrelation__lag_7  partial_autocorrelation__lag_8 |
| Permutation entropy | permutation_entropy__dimension_3__tau_1  permutation_entropy__dimension_4__tau_1  permutation_entropy__dimension_5__tau_1  permutation_entropy__dimension_6__tau_1  permutation_entropy__dimension_7__tau_1 |
| Quantile | quantile__q_0.1  quantile__q_0.2  quantile__q_0.3  quantile__q_0.8  quantile__q_0.9 |
| Range count | range_count__max_1__min_-1 |
| Ratio beyond r sigma | ratio_beyond_r_sigma__r_0.5  ratio_beyond_r_sigma__r_1  ratio_beyond_r_sigma__r_1.5  ratio_beyond_r_sigma__r_2  ratio_beyond_r_sigma__r_2.5  ratio_beyond_r_sigma__r_3  ratio_beyond_r_sigma__r_5 |
| Skewness | skewness |
| Spkt welch density | spkt_welch_density__coeff_2  spkt_welch_density__coeff_5  spkt_welch_density__coeff_8 |
| Time reversal asymmetry statistic | time_reversal_asymmetry_statistic__lag_1  time_reversal_asymmetry_statistic__lag_2  time_reversal_asymmetry_statistic__lag_3 |

## APG signal segment characteristics

In this study, APG timing subsegment used the same feature processing method as PPG and finally got 190 features. Table 3 shows the final 190 features obtained.

**Supplementary Table 3.** A total of 190 features were obtained for each APG timing subsegment. Note, CWT stands for Continuous wavelet transform, FFT stands for Fast Fourier transform.

| **characterization methods** | **features** |
| --- | --- |
| Absolute energy | absolute_sum_of_changes |
| Aggregation autocorrelation | agg_autocorrelation__f_agg_"var"__maxlag_40 |
| Aggregation linear trend | agg_linear_trend__attr_"intercept"__chunk_len_10__f_agg_"min"  agg_linear_trend__attr_"intercept"__chunk_len_10__f_agg_"var"  agg_linear_trend__attr_"intercept"__chunk_len_5__f_agg_"min"  agg_linear_trend__attr_"intercept"__chunk_len_5__f_agg_"var"  agg_linear_trend__attr_"intercept"__chunk_len_50__f_agg_"max"  agg_linear_trend__attr_"stderr"__chunk_len_10__f_agg_"max"  agg_linear_trend__attr_"stderr"__chunk_len_10__f_agg_"mean"  agg_linear_trend__attr_"stderr"__chunk_len_10__f_agg_"min"  agg_linear_trend__attr_"stderr"__chunk_len_10__f_agg_"var"  agg_linear_trend__attr_"stderr"__chunk_len_5__f_agg_"mean"  agg_linear_trend__attr_"stderr"__chunk_len_5__f_agg_"min"  agg_linear_trend__attr_"stderr"__chunk_len_5__f_agg_"var"  agg_linear_trend__attr_"stderr"__chunk_len_50__f_agg_"max"  agg_linear_trend__attr_"stderr"__chunk_len_50__f_agg_"var" |
| Approximate entropy | approximate_entropy__m_2__r_0.7  approximate_entropy__m_2__r_0.9 |
| Autoregressive coefficient | ar_coefficient__coeff_1__k_10  ar_coefficient__coeff_2__k_10  ar_coefficient__coeff_3__k_10  ar_coefficient__coeff_4__k_10  ar_coefficient__coeff_5__k_10  ar_coefficient__coeff_6__k_10  ar_coefficient__coeff_7__k_10  ar_coefficient__coeff_8__k_10 |
| Augmented Dickey-Fuller | Augmented_dickey_fuller__attr_"pvalue"__autolag_"AIC"  augmented_dickey_fuller__attr_"teststat"__autolag_"AIC" |
| Autocorrelation | autocorrelation__lag_1  autocorrelation__lag_2  autocorrelation__lag_3  autocorrelation__lag_4  autocorrelation__lag_5  autocorrelation__lag_6  autocorrelation__lag_7  autocorrelation__lag_8  autocorrelation__lag_9 |
| Benford correlation | benford_correlation |
| Binned entropy | binned_entropy__max_bins_10 |
| C3 | c3__lag_1  c3__lag_2  c3__lag_3 |
| Change quantiles | change_quantiles__f_agg_"mean"__isabs_False__qh_0.2__ql_0.0  change_quantiles__f_agg_"mean"__isabs_False__qh_0.4__ql_0.2  change_quantiles__f_agg_"mean"__isabs_False__qh_0.6__ql_0.4  change_quantiles__f_agg_"mean"__isabs_False__qh_0.8__ql_0.4  change_quantiles__f_agg_"mean"__isabs_False__qh_0.8__ql_0.6  change_quantiles__f_agg_"mean"__isabs_False__qh_1.0__ql_0.6  change_quantiles__f_agg_"mean"__isabs_True__qh_0.2__ql_0.0  change_quantiles__f_agg_"mean"__isabs_True__qh_0.4__ql_0.0  change_quantiles__f_agg_"mean"__isabs_True__qh_0.6__ql_0.0  change_quantiles__f_agg_"mean"__isabs_True__qh_0.6__ql_0.4  change_quantiles__f_agg_"mean"__isabs_True__qh_0.8__ql_0.0  change_quantiles__f_agg_"mean"__isabs_True__qh_0.8__ql_0.4  change_quantiles__f_agg_"mean"__isabs_True__qh_0.8__ql_0.6  change_quantiles__f_agg_"mean"__isabs_True__qh_1.0__ql_0.0  change_quantiles__f_agg_"mean"__isabs_True__qh_1.0__ql_0.2  change_quantiles__f_agg_"mean"__isabs_True__qh_1.0__ql_0.4  change_quantiles__f_agg_"mean"__isabs_True__qh_1.0__ql_0.6  change_quantiles__f_agg_"mean"__isabs_True__qh_1.0__ql_0.8  change_quantiles__f_agg_"var"__isabs_False__qh_0.2__ql_0.0  change_quantiles__f_agg_"var"__isabs_False__qh_0.4__ql_0.0  change_quantiles__f_agg_"var"__isabs_False__qh_0.6__ql_0.0  change_quantiles__f_agg_"var"__isabs_False__qh_0.6__ql_0.4  change_quantiles__f_agg_"var"__isabs_False__qh_0.8__ql_0.0  change_quantiles__f_agg_"var"__isabs_False__qh_0.8__ql_0.4  change_quantiles__f_agg_"var"__isabs_False__qh_0.8__ql_0.6  change_quantiles__f_agg_"var"__isabs_False__qh_1.0__ql_0.0  change_quantiles__f_agg_"var"__isabs_False__qh_1.0__ql_0.2  change_quantiles__f_agg_"var"__isabs_False__qh_1.0__ql_0.4  change_quantiles__f_agg_"var"__isabs_False__qh_1.0__ql_0.6  change_quantiles__f_agg_"var"__isabs_False__qh_1.0__ql_0.8  change_quantiles__f_agg_"var"__isabs_True__qh_0.2__ql_0.0  change_quantiles__f_agg_"var"__isabs_True__qh_0.4__ql_0.0  change_quantiles__f_agg_"var"__isabs_True__qh_0.4__ql_0.2  change_quantiles__f_agg_"var"__isabs_True__qh_0.6__ql_0.0  change_quantiles__f_agg_"var"__isabs_True__qh_0.6__ql_0.2  change_quantiles__f_agg_"var"__isabs_True__qh_0.6__ql_0.4  change_quantiles__f_agg_"var"__isabs_True__qh_0.8__ql_0.0  change_quantiles__f_agg_"var"__isabs_True__qh_1.0__ql_0.0  change_quantiles__f_agg_"var"__isabs_True__qh_1.0__ql_0.2  change_quantiles__f_agg_"var"__isabs_True__qh_1.0__ql_0.4  change_quantiles__f_agg_"var"__isabs_True__qh_1.0__ql_0.6  change_quantiles__f_agg_"var"__isabs_True__qh_1.0__ql_0.8 |
| Cid ce | cid_ce__normalize_False  cid_ce__normalize_True |
| Count above | count_above__t_0 |
| Count above mean | count_above_mean |
| Count below | count_below__t_0 |
| Count below mean | count_below_mean |
| FFT aggregated | fft_aggregated__aggtype_"centroid" |
| FFT coefficients | fft_coefficient__attr_"abs"__coeff_0  fft_coefficient__attr_"abs"__coeff_7  fft_coefficient__attr_"abs"__coeff_9  fft_coefficient__attr_"abs"__coeff_10  fft_coefficient__attr_"abs"__coeff_12  fft_coefficient__attr_"abs"__coeff_18  fft_coefficient__attr_"abs"__coeff_21  fft_coefficient__attr_"abs"__coeff_23  fft_coefficient__attr_"abs"__coeff_25  fft_coefficient__attr_"abs"__coeff_26  fft_coefficient__attr_"abs"__coeff_28  fft_coefficient__attr_"abs"__coeff_29  fft_coefficient__attr_"abs"__coeff_30  fft_coefficient__attr_"abs"__coeff_31  fft_coefficient__attr_"abs"__coeff_32  fft_coefficient__attr_"abs"__coeff_33  fft_coefficient__attr_"abs"__coeff_34  fft_coefficient__attr_"abs"__coeff_35  fft_coefficient__attr_"abs"__coeff_36  fft_coefficient__attr_"abs"__coeff_37  fft_coefficient__attr_"abs"__coeff_38  fft_coefficient__attr_"abs"__coeff_39  fft_coefficient__attr_"abs"__coeff_40  fft_coefficient__attr_"abs"__coeff_42  fft_coefficient__attr_"abs"__coeff_43  fft_coefficient__attr_"abs"__coeff_44  fft_coefficient__attr_"abs"__coeff_46  fft_coefficient__attr_"abs"__coeff_47  fft_coefficient__attr_"abs"__coeff_48  fft_coefficient__attr_"abs"__coeff_49  fft_coefficient__attr_"abs"__coeff_50  fft_coefficient__attr_"abs"__coeff_51  fft_coefficient__attr_"abs"__coeff_52  fft_coefficient__attr_"abs"__coeff_53  fft_coefficient__attr_"abs"__coeff_54  fft_coefficient__attr_"abs"__coeff_56  fft_coefficient__attr_"abs"__coeff_58  fft_coefficient__attr_"abs"__coeff_61  fft_coefficient__attr_"abs"__coeff_66  fft_coefficient__attr_"abs"__coeff_75 |
| Fourier entropy | fourier_entropy__bins_10  fourier_entropy__bins_100  fourier_entropy__bins_3  fourier_entropy__bins_5 |
| Friedrich coefficients | friedrich_coefficients__coeff_1__m_3__r_30  friedrich_coefficients__coeff_2__m_3__r_30  friedrich_coefficients__coeff_3__m_3__r_30 |
| Kurtosis | kurtosis |
| Large standard deviation | large_standard_deviation__r_0.15000000000000002 |
| Lempel-Ziv complexity | lempel_ziv_complexity__bins_10  lempel_ziv_complexity__bins_100  lempel_ziv_complexity__bins_5 |
| Matrix Profile | matrix_profile__feature_"75"__threshold_0.98  matrix_profile__feature_"mean"__threshold_0.98  matrix_profile__feature_"median"__threshold_0.98 |
| Max langevin fixed point | max_langevin_fixed_point__m_3__r_30 |
| Maximum | maximum |
| Mean absolute change | mean_abs_change |
| Median | median |
| Minimum | minimum |
| Number crossing m | number_crossing_m__m_0  number_crossing_m__m_-1 |
| Number CWT peaks | number_cwt_peaks__n_1 |
| Number peaks | number_peaks__n_1  number_peaks__n_10  number_peaks__n_3  number_peaks__n_5  number_peaks__n_50 |
| Partial autocorrelation | partial_autocorrelation__lag_1  partial_autocorrelation__lag_2  partial_autocorrelation__lag_4 |
| Permutation entropy | permutation_entropy__dimension_3__tau_1  permutation_entropy__dimension_4__tau_1  permutation_entropy__dimension_5__tau_1  permutation_entropy__dimension_6__tau_1  permutation_entropy__dimension_7__tau_1 |
| Quantile | quantile__q_0.1  quantile__q_0.3  quantile__q_0.4  quantile__q_0.6  quantile__q_0.7  quantile__q_0.8  quantile__q_0.9 |
| Range count | range_count__max_1__min_-1  range_count__max_1000000000000.0__min_0 |
| Ratio beyond r sigma | ratio_beyond_r_sigma__r_0.5  ratio_beyond_r_sigma__r_1  ratio_beyond_r_sigma__r_1.5  ratio_beyond_r_sigma__r_2  ratio_beyond_r_sigma__r_2.5  ratio_beyond_r_sigma__r_3 |
| Skewness | skewness |
| Spkt welch density | spkt_welch_density__coeff_2  spkt_welch_density__coeff_5  spkt_welch_density__coeff_8 |
| Symmetry looking | symmetry_looking__r_0.05 |
| Time reversal asymmetry statistic | time_reversal_asymmetry_statistic__lag_1  time_reversal_asymmetry_statistic__lag_2  time_reversal_asymmetry_statistic__lag_3 |
