# Supplementary figures and images for: Blood pressure stratification using photoplethysmography and light gradient boosting machine
Source: Front Physiol. 2023 Feb 20;14:1072273. doi: 10.3389/fphys.2023.1072273 (PMC9986584; doi:10.3389/fphys.2023.1072273)

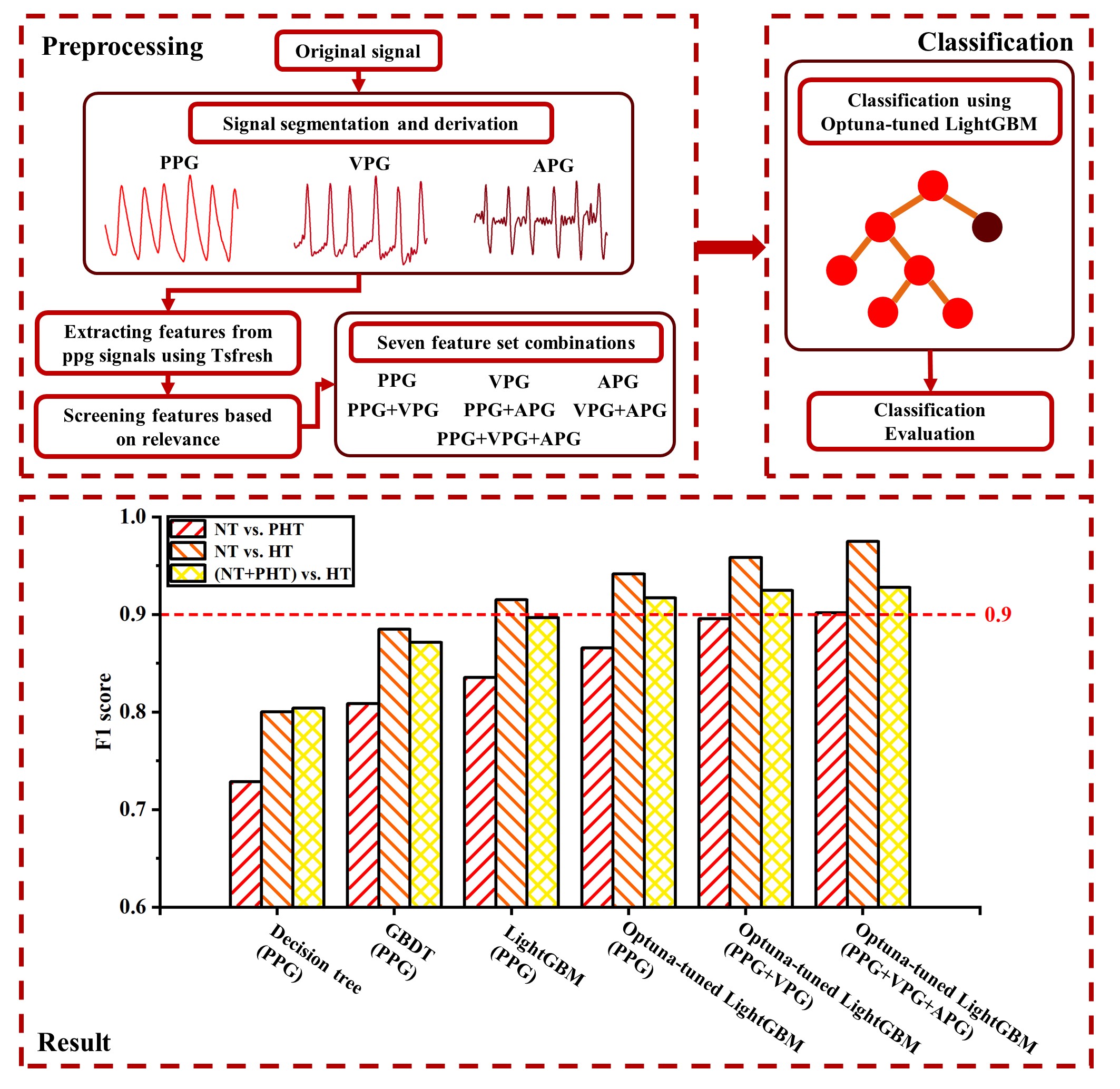

Supplement: Supplementary file 2 [file Image1.JPEG]
